# Winter is coming: How laypeople think about different kinds of needs

**DOI:** 10.1371/journal.pone.0294572

**Published:** 2023-11-27

**Authors:** Alexander Max Bauer, Jan Romann, Mark Siebel, Stefan Traub

**Affiliations:** 1 Department of Philosophy, University of Oldenburg, Oldenburg, Germany; 2 Faculty of Technology, University of Applied Sciences Emden/Leer, Emden, Germany; 3 Department of Economics, Helmut-Schmidt-University, Hamburg, Germany; University of Pretoria, SOUTH AFRICA

## Abstract

Needs play a key role in many fields of social sciences and humanities, ranging from normative theories of distributive justice to conceptions of the welfare state. Over time, different conceptions of what counts as a need (i. e., what is considered a normatively relevant need) have been proposed. Many of them include (in one way or the other) needs for survival, decency, belonging, and autonomy. Little work has been done on how these kinds of needs are evaluated in terms of their significance for distributive justice. To begin closing this gap, we investigate the role of the four aforementioned kinds of needs for impartial observers. We do so in two empirical studies. The first study asks participants to evaluate the importance of each of the four kinds of needs separately. We find that different levels of importance are attributed to the kinds of needs, which places them in a hierarchy. The second study asks participants to make distributive decisions. Results further support the hierarchy found in the first study and, additionally, reveal that participants tend to make coherent allocation decisions.

## Introduction

Imagine you were living in a cottage heated exclusively with firewood. Spring has given way to summer, summer has given way to autumn—and temperatures are starting to fall. Winter is coming, and, unfortunately, you are short on firewood. Now imagine that without additional firewood it would get so cold in your hut that you would probably become life-threateningly ill.

In this case, your physical integrity—something that pretty much all authors can agree counts as a basic need—is seriously threatened. Such needs have played a role in philosophy since antiquity (see, e. g., [1], who interprets Aristotle’s reflections on ἀναγκαῖον in his *Metaphysics* as related to basic needs; [2, 1015a20–1015b15]), they feature in the *Acts of Luke*, when the Christian community is described (see [3, p. 141f.] and [4, p. 302f.]), and they have repeatedly been emphasized in the history of thought (famously, e. g., by [5]). In the last century, psychology (see, e. g., [6, 7]) and philosophy (see, e. g., [8–10]; for overviews see [1, 11–13]), among some other fields, found new interest in the topic (for perspectives from philosophy, psychology, sociology, political science, and economics, see [14]). Also, needs have gained new weight as a cornerstone of the welfare state (see, e. g., [15]).

Authors have developed countless theories on what counts as a basic need. Few things obtain such a unanimous approval as physical integrity as described above. In the course of this paper, we identify a *hierarchy* of four kinds of needs that recur in the literature: survival, decency, belonging, and autonomy. We ask whether these needs are perceived as having different degrees of importance (first indications that this might indeed be the case are provided by a few studies, see [16, 17]). To test this, we designed and conducted two empirical studies.

The first study elicits absolute need evaluations. Here, participants were first given short vignettes presenting our four types of needs in a hypothetical context. Each vignette was introduced with an illustration of the hypothetical situation, drawn by artist Douwe Dijkstra. Then, for each of them, participants had to indicate how important they consider the need in question to be. We find that different levels of importance are attributed to the kinds of needs, effectively placing them in a clear hierarchy.

The second study sheds light on relative need evaluations. Here, too, participants were first familiarized with vignettes of the four kinds of needs and the corresponding illustrations. They then were presented with cases showing two people. Cases varied as to what kind of need the two people experience. The participants’ task was to distribute a scarce good between these two hypothetical people as impartial decision-makers. As an additional within-participants variation, we altered whether both persons contributed equally or unequally to the amount available for distribution. This way, we investigate whether productivity has an effect on the decisions. Our findings further support the hierarchy we found in the first study. Moreover, productivity has an effect on participants’ decisions. In addition, we are able to observe that our participants’ need evaluations are coherent in terms of adding up.

## Literature review

As recent research has pointed out (see, e. g., [18, 19]), needs are not only relevant for normative reflections, but have also been the subject of numerous empirical studies. Think, for example, of early empirical social choice (see, e. g., [20]) and what followed from it (for a review, see [21]). At least since the beginnings of motivational psychology (see, e. g., [22]) they are also to be found in the field of psychology (for a review, see [23]). Population surveys have also revealed that basic needs play a role in people’s ideas of justice (see, e. g., [24, 25]). Incentivized economic experiments have shown that needs influence the subjects’ decisions (see, e. g., [26, 27]). There were also attempts to bring real needs into the laboratory (see, e. g., [28]). Hence, our studies and their implications regarding the concept of need potentially touch on many different fields of research.

The concept of need has been defined in different ways. Generally, needs may be attributed by the locution “*S* needs *x* in order to Φ”. In the context of justice, the expression at *S* typically refers to persons, but we may equally think of households, companies, and so forth. The term at *x* designates the object of a need, viz., the thing needed. This can be a material resource, but also other goods, such as personal relationships. The expression at Φ stands for the goal to be achieved with the object of the need, which may be an action, a status, an opportunity, and so forth. In any case, by claiming that *S* needs *x* in order to Φ, it is stated that *x* is *necessary* for *S* to achieve Φ.

A need claim can be *purely instrumental*, such as “She needs a hammer in order to knock in the nail”. Such a claim is morally neutral in itself; its moral relevance depends, among other things, on the moral relevance of the goal involved. Many authors differentiate such instrumental needs from *categorical* (absolute or intrinsic) ones. Categorical needs are distinguished from mere wants, wishes, or desires—either through some objective criterion (see, e. g., [29–32]) or through some inter-subjective process (see, e. g., [9, 10, 33]). Furthermore, they are assumed to bear a normative force since their aim is regarded as something that ought to be realized. In other words, they are taken to be “necessary, indispensable, or inescapable, at least with respect to some important goals” [11, par. 37]. The overarching goal most commonly used to characterize categorical needs is avoidance of harm, or in positive terms, living a decent life (see, i. a., [8, 9, 30, 34–37]).

There are quite a few distinctions between categorical needs to be found in the literature (note that, in the course of history, a variety of lists of such needs have been suggested, specifying what counts as a basic need, e. g., [38–40]; others have opposed these attempts to draw up concrete lists and have instead made *categorizations* of basic needs, e. g., [10]). Frequently, they are categorized in form of a hierarchy that is based on the priority of satisfying these needs. The most noted hierarchy is, arguably, the one of Maslow (see, e. g., [6]), which is part of his psychological Motivation Theory. It is usually represented as a pyramid constituted of, from the bottom up, *physiological* needs, *safety* needs, *social* needs, *esteem* needs, and *self-actualisation* needs. Alderfer (see, e. g., [7, 41]) modified Maslow’s account by differentiating between *existence* needs, *relatedness* needs, and *growth* needs, developing the “Existence, Relatedness, and Growth Need Questionnaire” that was used, e. g., by [42] (note also [22], who considers safety, belonging, and esteem). Roughly, existence needs combine Maslow’s physiological and safety needs, relatedness needs combine social and esteem needs, and growth needs can be identified with self-actualisation needs. Alderfer’s hierarchy thus resembles the distinction between *vital* needs, *social* needs, and *agency* needs by Hamilton [10, 43].

Moreover, the idea of multidimensional poverty indices can be interpreted as representing different kinds of needs (on the relationship between poverty and need, see [44], for multidimensional measurement of need-based justice, see [45, 46]). In medical and care contexts there is also use of differentiations between needs (see, e. g., [47–50]).

Assigning top priority to needs directed at the very existence, such as food, shelter and sleep, is at the heart of the Basic Needs Approach [51, 52]. Moreover, it is a common thread in philosophical accounts (e. g., [36, 37, 39]), probably because such needs promise the highest objectivity and, due to their vital importance, the greatest moral significance. Nonetheless, only a minority of scholars put their focus on them (e. g., [53]). It is much more common to hold that there are basic needs beyond the biological minimum not to be ignored just because existence comes first. Such needs are often derived by asking what is necessary to function in social groups (e. g., [8, 37, 39]) or our ability to function as human agents (e. g., [54–57]), as Brock and Miller summarize [11]. Along these lines, Miller argues that “[h]uman beings are social as well as biological creatures” and takes “basic needs” to be the “conditions for a decent human life in any society” while “societal needs” are the additional “requirements for a decent life in the particular society to which the person belongs” [58].

Regardless of whether those needs are aptly named basic or not, we want to integrate them into our study. Following Alderfer’s distinction between relatedness needs and growth needs, we divide them into needs for social belonging and needs for autonomy, such as the need for self-actualization by creative work. The latter echoes Hamilton’s agency needs, which include autonomy, recognition, and creative expression. In line with most of the literature, we assume that social belonging and autonomy needs possess lower priority than needs for mere survival. Furthermore, under the general heading of needs for a decent life, we interpose the need not to feel cold. Of course, not feeling cold is merely one aspect of a decent life. But since the concept of a “decent life” is too broad and not feeling cold is a central and vivid aspect of a decent life, we make use of this description. In this sense, we take not feeling cold to be a paradigm ingredient of a decent life. In decreasing priority, we thus distinguish four kinds of needs:

- needs for mere survival,
- needs for a decent life,
- needs for social belonging, and
- needs for autonomy.

Those kinds are not to be understood as mutually exclusive. As Hamilton has already put it (regarding his own typology): “the boundaries between the […] categories are necessarily porous” [10, p. 23].

While there are no studies that explicitly test the philosophical considerations outlined above, empirical tests of Maslow’s theory emerged quickly. A large number of these originated before the 1980s in the field of organisational psychology. Most of these studies only find marginal support for his theory (see, e. g., [59–64]; for overviews see [65, 66]).

Methodologically, the aforementioned studies often assess satisfaction scores or need strength scores of participants in a working environment, many making use of the “Porter Need Satisfaction Questionnaire” [67]. In retrospect, it is astonishing that a large part of research on something as fundamental as basic human needs was primarily conducted not in a general setting but restricted to the workspace. We chose another path by utilizing hypothetical vignettes to analyze the importance ascribed to different kinds of needs. This takes place, in part, in the wake of the growing experimental social choice literature which goes back to the investigations of distributive choices (e. g., by [20, 68]). For overviews see, e. g., [21, 69–71]. Vignettes have been used famously, e. g., by [72, 73], or recently in experimental philosophy, e. g., by [74, 75]. For use in justice research see, i. a., [76–78]; for use in need contexts, see, e. g., [79]. Insightful reflections on using empirical studies to investigate justice evaluations are to be found in [80–82]. Also see [83, 84]. Gaertner and Schokkert carefully address methodological issues regarding empirical social choice using laboratory and survey experiments (see [21]). They also discuss the pros and cons of (not) using monetary incentives in order to elicit justice and distributional preferences. While game-theoretic experiments make predictions about *actual* behavior, which is to some extent driven by self-interest, they underline that the aim of empirical social choice is to derive information about people’s *norms*. As Konow puts it, vignette studies “provide a contextual richness that is better suited” than incentivized experiments to study fairness judgments embedded in real social institutions [71, p. 109]. Another argument against using financial incentives in our study are experimenter demand effects (EDEs), i. e., participants might also be induced by payoffs to respond in a way that confirms the experimenter’s hypothesis if it is known [85]. [86] shows that hypothetical survey experiments are robust against EDEs. Most importantly, however, EDEs are reduced by minimizing interaction between experimenter and participants and not announcing a hypothesis (see [87]), as in our study.

In this strand of literature, there are only very few to no experiments on distributive decisions implementing different kinds of needs. Some vary contexts that might in turn be interpreted as representing different spheres of need. These variations are rather extreme, though, so that comparability amongst them is at risk: For example, some use food and medicine in a catastrophy and non-catastrophy variation [88], others use trade-offs between “helping a handicapped person or teaching intelligent children”, giving “financial aid to starving people in Subsaharan Africa versus an environmental programme in the home country” of a participant, or “a set of measures for rapid economic reconstruction at the expense of some basic human rights and a slower economic recovery going hand in hand with a full restoration of these human rights” [89, p. 630] (also see, i. a., [69, 89–91]). Here, the differences between contexts are so large that it is impossible to tell whether differences found between them are due to the different kinds of needs or due to other factors in the varying contexts. An exception is [17], who systematically varied the kinds of needs, but did not find any influence of the different kinds in a hypothetical distribution task (prior to this, [45, 46] argued for the importance of differentiating kinds of need, originally in the context of measuring need-based distributive justice, but also—on a side-note—extending to empirical research on need-based distributive justice).

In summary, despite the extensive research inspired by Motivation Theory, there seems to be little to no systematic research on different kinds of needs in the context of distributive justice. Those few that touch upon this topic vary kinds of needs rather unsystematically. We therefore want to study the impact of different kinds of needs on distributive decisions made by impartial observers. The main contributions of this paper to the existing social-choice literature discussed above are as follows: In Study 1, using absolute evaluations of the importance of the four kinds of needs, we show that there is a clear hierarchy among them. In Study 2, using the relative ratings, we confirm the hierarchy among the kinds of needs and show how accountability in terms of productivity differences interacts with the relative evaluations of need.

Moreover, in the further course of this paper, we will also investigate how coherent the hypothetical decisions of our participants are. Our understanding of coherence is derived from the philosophical coherence theory of justification. According to this theory, a system of beliefs is more justified the more its elements cohere, where coherence does not only mean consistency but also hanging or fitting together. There is a debate on the exact relations governing coherence, but it is generally accepted that *entailment* positively contributes to it, whereas *contradiction* has a negative influence (cf. [92–94]). For example, if *A* and *B* imply *C*, then there is, *ceteris paribus*, more coherence than in cases where *A* and *B* contradict *C*. When it comes to beliefs about magnitudes, this means that values should add up. Consider a person who thinks that Anne is 2 inches taller than Ben, and Ben 3 inches taller than Clare, but also thinks that Anne is 4 inches taller than Clare. Then her beliefs fit together less than the beliefs of a person who agrees with the first two assumptions but additionally believes that Anne is 5 inches taller than Clare. For “Anne is 2 inches taller than Ben” and “Ben is 3 inches taller than Clare” contradict “Anne is 4 inches taller than Clare”, whereas they imply “Anne is 5 inches taller than Clare”. In this spirit, we will examine whether the differences in priority assigned to our four kinds of needs add up.

## Study 1

### Design

In our first study, participants are asked to evaluate the importance of our four kinds of needs in absolute terms. We take a two-stage approach: First, participants are given an overview of the different kinds of needs. Second, they evaluate each kind separately and on a scale that relates directly to the kinds’ importance. In order to control for ordering effects, participants are randomly assigned ex-ante to one of 24 possible sequences of the four kinds of needs. Note, however, since participants who can not answer more than one control question (as explained below) are excluded from further participation, the number of observations per sequence is not exactly balanced ex-post.

**Fig 1:**
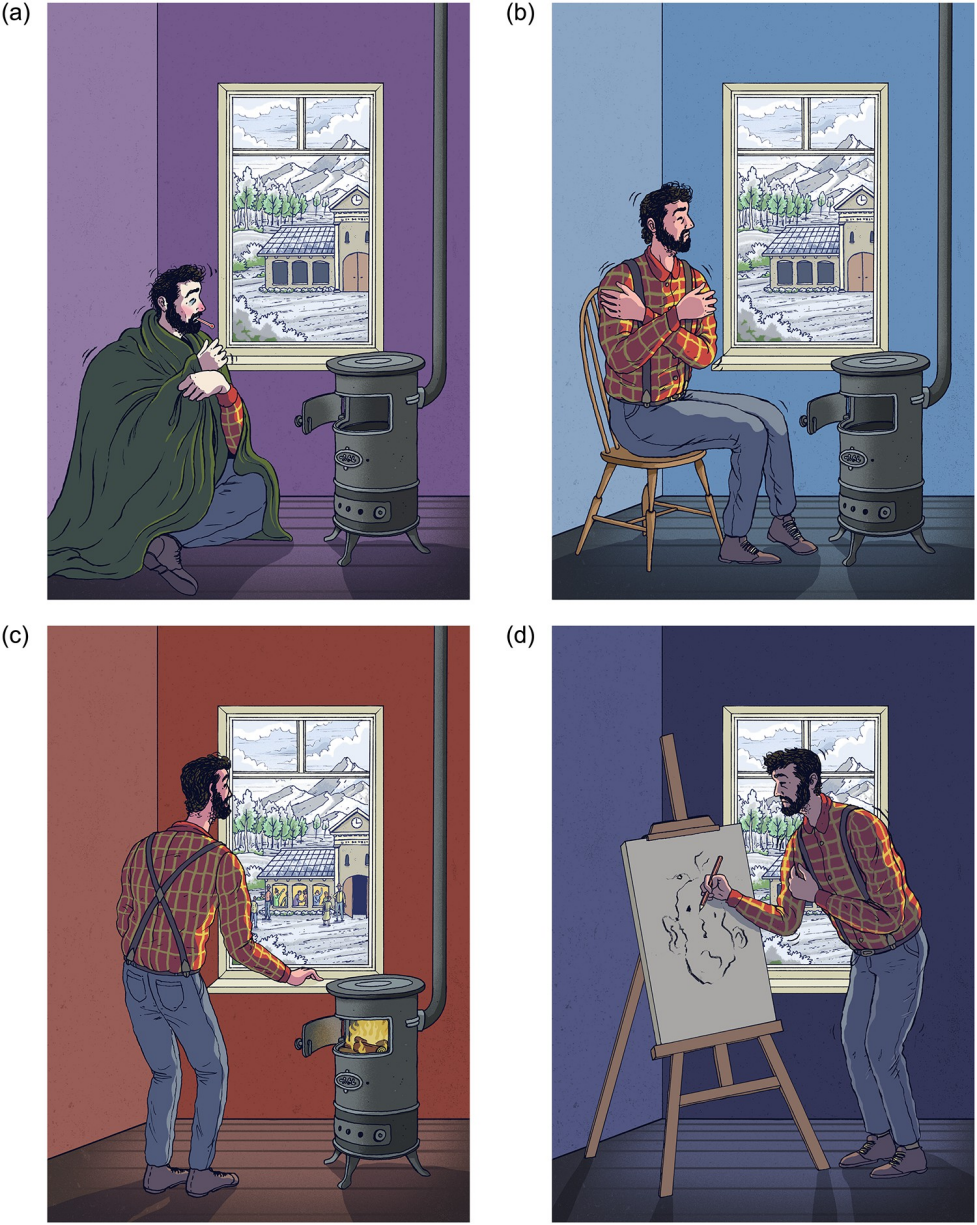
Illustrations presented to participants. 1a shows the Survival Need, 1b the Decency Need, 1c the Belonging Need, and 1d the Autonomy Need. Republished from the original study under a CC BY license, with permission from Douwe Dijkstra, original copyright 2021.

In the first stage, we ask them to imagine four people with different names (that are randomly drawn from a pool of common German surnames). All four are in need of firewood for different reasons respectively. In all four instances, the need is not merely instrumental, but is regarded as having normative weight, as has been shown in the Literature review. Below each other, the four people and their respective needs are introduced with an illustration (see Fig 1) and a short vignette (see S1 Appendix for wording).

In accordance with the classification presented in the Literature review, participants are told that a person can need the wood either for survival, decency, belonging, or autonomy. We present four short vignette texts (the vignette’s scenario is adapted from [19]), each exemplifying one of those kinds of needs: A person can need the wood to

- survive the upcoming winter. This means that if they receive less wood than they need, it will become so cold in their hut that they are very likely to fall life-threateningly ill.
- avoid feeling cold in the upcoming winter. The members of their community agree that one cannot live in dignity if one has to feel cold. If they receive less wood than they need, it becomes unacceptably cold in their hut.
- be able to participate regularly in the social life of their community in the upcoming winter since it is common practice to meet in the community center and everyone is expected to bring wood to heat it. If they receive less than they need, they will not be able to participate regularly.
- be able to plan their leisure time autonomously, since they usually use their spare time to create art in their studio, which is heated with wood. If they receive less than they need, they won’t be able to use their studio regularly.

Note that the English wording “not freezing”, and perhaps also “not feeling cold”, is difficult to distinguish from “not catching a life-threatening illness”. However, the German wording “nicht frieren” clearly refers to a different matter than “nicht lebensbedrohlich erkranken” because “frieren” (feeling cold) rarely results in a life-threatening illness; it usually refers to an unpleasant but not severe state.

Following this introduction, in the second stage, the kinds of needs are presented to participants on four separate screens. Here, they are shown the full-sized illustration with a single caption beneath it describing the kind of need once again as follows. Note that the denominations used by us (“Survival”, “Decency”, “Belonging“, and “Autonomy”) are not shown to participants.

- **Survival:** “*A* needs the wood to make sure to survive the upcoming winter.”
- **Decency:** “*B* needs the wood in order not to feel cold in the upcoming winter.”
- **Belonging:** “*C* needs the wood to be able to participate regularly in the social life of his community in the upcoming winter.”
- **Autonomy:** “*D* needs the wood to be able to use his studio regularly in the upcoming winter”.

On top of each screen, they are told that they will have to indicate how important they deem the kind of need shown on the page. On the bottom, they are asked how much the person needs the wood in the case in question. They have to give their answer on a Likert scale from 1 (“doesn’t need the wood at all”) to 7 (“absolutely needs the wood”) (see S1 Appendix for the instructions of Study 1). That is, participants indicate how important they consider the satisfaction of the abstract need through a concrete material good. Their evaluation is thus conditional on the situation and the material good.

We have purposely chosen this setting although it is remote from the lifeworld of our participants. The reason is that they are meant to act as social planners making theoretical decisions without influence from their personal situation. We thus need a vignette that is vivid but not part of their everyday life. A former study has shown that the given vignette seems to be well-suited for this purpose (see [19]).

### Procedure

The study was programmed in oTree (see [95]) and was conducted online in February 2021 with a sample size of *n* = 100. Participants were recruited by the professional market research institute respondi for a fee, where they were randomly sampled from respondi’s online access panel, stratified by the three characteristics gender, age, and equivalent household net income (see S1 Table). The data provider has subjected itself to strict ethical guidelines and is certified according to ISO 20252. Participants must actively opt into the panel to then take part in specific surveys voluntarily. Since our study is an anonymous standard survey that only uses hypothetical vignettes and assesses uncritical opinions, no additional approval was sought from the ethics commission. As suggested, e. g., by the United States’ *Federal Policy for the Protection of Human Subjects*, to ensure informed consent, participants were told at the beginning of each study that the survey at hand is part of a research project, that participation is voluntary, and that they can drop out at any time. They were notified how long the survey would approximately take and that its purpose is to assess personal opinions and judgments. They were also informed that their answers will be analyzed and that all data will be stored in an anonymous format so that no participant can be identified (see S1 and S4 Appendices). They were informed beforehand about their compensation by the panel provider.

At the beginning of the study, they were greeted with a welcome message (see S1 Appendix). Thereafter, participants had to evaluate the four kinds of needs as described in Design (within-participants design). After this task, three control questions were asked to ensure that participants read the vignettes and instructions carefully (see S2 Appendix). As had been announced beforehand, only those participants who passed at least two of our control questions were compensated and included in our analysis. Failing more than one question led to an immediate exclusion of the participant from the survey. The 100 participants who answered two or three questions correctly were given a sociodemographic questionnaire asking for their age, gender, household net income, political orientation, and sensitivity to cold (see S3 Appendix). They were paid a flat fee of 4.15 euros for approximately 15 minutes of their time. 31 participants were excluded from the study after failing to pass control questions.

Failure rates indicate that our first question was failed more often than the second and third question (Question 1: 41 of 131 (31.30%), Question 2: 36 of 131 (27.48%), Question 3: 27 of 131 (20.61%)). Using *χ*^2^ tests, we found that the excluded participants did not diverge from the remaining participants with regard to age and income at a significance level of 5%. There is, however, a significant difference at the 1% level between men and women when it comes to failure rates (Age: *χ*^2^ = 3.787, *p* = 0.436, Income: *χ*^2^ = 8.511, *p* = 0.075, Gender: *χ*^2^ = 7.238, *p* = 0.007).

### Working hypothesis

In light of the theoretical work we looked at in our Literature review, we suspect that participants ascribe some importance to all four kinds of needs. We also suspect that the importance of the kinds of needs that are more basic in theory is evaluated higher on average than the importance of those that are less basic in theory. More specifically, in line with psychological Motivation Theory (see, e. g., [6, 41]) and philosophical considerations (see, e. g., [36, 37, 39]), we hypothesize that physiological needs (Survival, Decency) receive higher importance ratings than social needs (Belonging) and individual needs (Autonomy). If we look at the means (*M*) of importance ratings, we can state as **Hypothesis 1 (Hierarchy in *M*)**:

$$\begin{array}{c} M_{Survival}>M_{Decency}>M_{Belonging}>M_{Autonomy}. \end{array}$$ (1)

### Results

**Fig 2:**
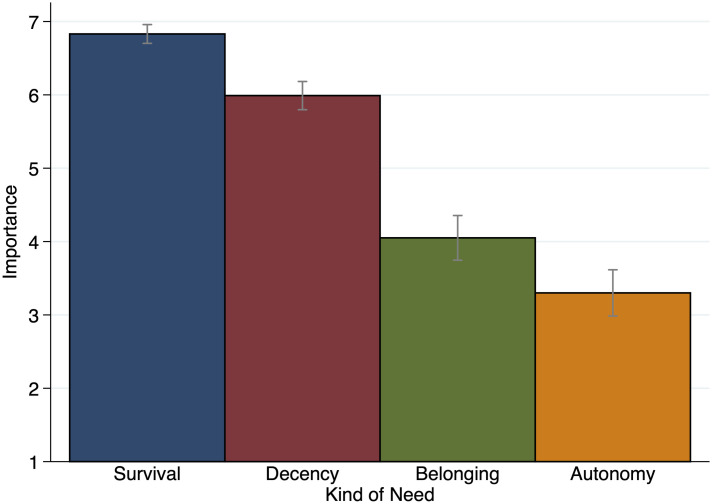
Mean importance ascribed to the four kinds of needs. The figure shows the mean importance ascribed to the four kinds of needs on a scale from 1 (“does not need the wood at all”) to 7 (“absolutely needs the wood”). *n* = 100.

As hypothesized, the importance of the four kinds of needs was rated quite differently, as shown in Fig 2 and Table 1 (note that for each need type, participants were also allowed to refuse to answer; therefore, there are only 99 observations for Decency and Belonging; further note that the same results are obtained for both the importance ratings in Table 1 and the predicted probabilities of the ordered-logit estimation in Table 2 when only including participants who answered all control questions correctly; *n* = 72 in Survival, Decency, and Autonomy; *n* = 71 in Belonging). The Survival Need scored highest with a mean rating of 6.830 (*σ* = 0.065, 95% CI [6.701, 6.959]), followed by the Decency Need with a mean rating of 5.990 (*σ* = 0.098, 95% CI [5.796, 6.184]). Third in line, after a notable drop, is the Belonging Need with a mean rating of 4.051 (*σ* = 0.155, 95% CI [3.743, 4.358]), followed by the Autonomy Need with a mean rating of 3.300 (*σ* = 0.160, 95% CI [2.982, 3.618]). Note that the scale is limited to the interval [1, 7]. One could assume that many participants choose the maximum value for Survival, and rightfully so, as 92 of 100 participants did. For Decency, only 31 did so. With this in mind, we performed pairwise Wilcoxon signed-rank tests to test for the equality of the distributions of importance evaluations between the kinds of needs, which are displayed (together with the respective mean differences) in the lower panel of Table 1. Thus, the comparison of the mean values and the distributions of the importance evaluations clearly confirms Hypothesis 1.

**Table 1:** Mean importance ascribed to kinds of needs and differences between them.

	Survival	Decency	Belonging	Autonomy
Mean	6.830	5.990	4.051	3.300
Std. Dev.	0.065	0.098	0.155	0.160
Decency	−0.840***			
Belonging	−2.779***	−1.939***		
Autonomy	−3.530***	−2.690***	−0.751***	

Upper panel: Mean of the ascribed importance and Standard Deviation. Lower panel: Mean differences and significance levels of a Wilcoxon matched-pairs signed-rank test on the equality of two distributions. Significance levels:

* *p* < 0.10,

** *p* < 0.05,

*** *p* < 0.01.

*n* = 100 in Survival and Autonomy, *n* = 99 in Decency and Belonging.

**Table 2:** Ordered logit estimation results.

	Survival	Decency	Belonging	Autonomy
Age	-0.009	0.004	-0.008	-0.014
{♯*years*}	(0.028)	(0.014)	(0.011)	(0.013)
Gender	-0.469	-0.389	0.235	-0.077
{0 = *female*, 1 = *male*}	(0.829)	(0.416)	(0.461)	(0.433)
Household Net Income	0.405e -4	-9.52e -6***	-4.31e -6	3.73e -6**
{*euros*}	(1.21e -4)	(3.55e -6)	(2.65e -6)	(1.78e -6)
Political Attitude	0.081	-0.014 ***	-0.004	-0.009 **
{1, …, 7}	(0.217)	(0.005)	(0.005)	(0.004)
Sensitivity to Cold	-0.150	-0.183	0.143	0.108
{1, …, 7}	(0.217)	(0.137)	(0.182)	(0.149)
*n*	100	99	99	100
Wald *χ*^2^	1.69	14.47 **	5.38	19.01 ***
Pseudo-*R*^2^	0.027	0.021	0.007.	0.010
Predicted Probabilities				
7 (“absolutely”)	91.94	31.31	7.19	5.96
	[91.23, 92.65]	[29.75, 32.87]	[6.84, 7.53]	[5.60, 6.32]
6	3.01	48.91	7.24	1.98
	[2.76, 3.26]	[48.35, 49.46]	[6.95, 7.54]	[1.88, 2.09]
5	2.02	10.95	25.53	12.91
	[1.84, 2.20]	[10.33, 11.57]	[24.90, 26.16]	[12.33, 13.49]
4	2.03	5.83	30.15	22.98
	[1.84, 2.21]	[5.33, 6.33]	[29.97, 30.34]	[22.46, 23.50]
3	1.01	3.01	10.89	20.18
	[0.91, 1.10]	[2.68, 3.33]	[10.59, 11.17]	[20.03, 20.32]
2	0	0	12.94	23.04
			[12.40, 13.48]	[22.30, 23.78]
1 (“not at all”)	0	0	6.06	12.94
			[5.72, 6.40]	[12.20, 13.67]

The table reports the results of an ordered logit estimation with robust standard errors by kind of need. Endogenous variable: importance evaluation ({1, …, 7}). Upper panel: first row: coefficients, second row: standard errors in parentheses. Significance levels:

* *p* < 0.10,

** *p* < 0.05,

*** *p* < 0.01.

Lower panel: predicted probabilities of the respective importance evaluation by kind of need, first row: means, second row: 95% confidence intervals.

The order in which the kinds of needs are displayed is randomized and nearly balanced, and therefore should not play a role in the mean importance evaluations. In Study 2, explained below, the absolute importance evaluation is collected using the same procedure as in Study 1, but only after the main task, the distribution decision. Pairwise comparisons of the evaluations of the same kinds of needs between the two studies using a Wilcoxon rank-sum test show no significant differences except for Autonomy, for which the evaluation in Study 2 is even worse than in Study 1 (Survival: *p* = 0.220, Decency: *p* = 0.366, Belonging: *p* = 0.677 Autonomy: *p* = 0.033). In Study 2, we also recorded the sequence in which the kinds of needs are presented and their evaluations are collected. Here, no significant differences are found if a need type is presented first or later except for Belonging, which is evaluated slightly more important if presented first (Wilcoxon rank-sum test, Survival: *p* = 0.873, Decency: *p* = 0.278, Belonging: *p* = 0.095, Autonomy: *p* = 0.934). Altogether these tests underscore the robustness of the results to ordering effects.

In Table 2, we present the results of an ordered logit estimation to test the robustness of the previous result with respect to the characteristics of the participants. From the top panel, we see that age and gender, as well as sensitivity to cold, do not correlate with the importance ratings of the kinds of needs. There is a significant correlation between household net income and importance ratings with respect to Decency (negative) and Autonomy (positive), where the latter correlation is unsurprising given that richer households are more likely to consider self-actualization important. Moreover, it is not surprising that more right-wing participants tend to value needs beyond Survival less (empirical studies show that right-wing survey participants believe more strongly in personal responsibility and are less in favor of income redistribution, see., e. g., [96, 97]). Overall, however, the explanatory power of personal characteristics for the evaluation of the four kinds of needs is very low.

The lower panel of Table 2 shows the probabilities of the respective importance evaluation computed from the ordered logit estimation separately for the four kinds of needs. It is evident that also when controlling for personal characteristics, survival needs are clearly evaluated as most important (almost 92% of participants choose the highest category, i. e., 7). Decency needs are also evaluated as very important, with the median and mode being category 6 (almost 49% of participants). Belonging needs show a significantly higher dispersion of evaluation, with the median and mode right in the middle category 4 (slightly more than 30%), i. e., participants are much less unanimous about the importance of Belonging. The greatest dispersion or dissent is found in Autonomy. Here, the median is category 3 (20%) and the mode is 4 (23%). Overall, the hierarchy of needs suggested by Hypothesis 1 is confirmed and, in addition, greater dissent among participants is evident for less important needs.

## Study 2

### Design

For our second study, we join the vignette of Study 1 with a vignette by Bauer and colleagues (see [19]). There, participants are asked to imagine two hypothetical persons. Their names are drawn randomly from a list of common German surnames. In the following, we simply refer to them as “Person A” and “Person B”. Person A and Person B are both in need of firewood. Their community allows them to chop wood in the community’s forest for a certain period of time, which is the only way for Person A and Person B to get firewood, since both have little money.

In the fashion of Study 1, we alter the vignette by adding the four different kinds of needs that Person A and Person B can experience. Participants are introduced to those four kinds at the study’s beginning. As in Study 1, each vignette (see S4 Appendix for wordings) is presented next to a picture that illustrates the kind of need in question (see Fig 1 again).

Subsequent to this introduction, participants are introduced to their task, which is to distribute an endogenously given number of logs—described as the total amount of wood both have chopped—among Person A and Person B in a way participants think to be most just. They are made aware that in doing so they will have to make trade-offs between Person A and Person B; the more wood they give to one person, the less they can give to the other. Additionally, we revealed that it would not be possible to completely meet the needs of both persons at the same time, as the available amount of wood was just about enough to completely cover the needs of one of the two persons; the other person would in that case end up empty-handed. In case a person receives less wood than they need, the person will suffer certain consequences, depending on the kind of need they experience. Participants were further informed that they would distribute the wood in advance without knowing exactly how cold the winter will actually be. This is why we describe the possible effects of the upcoming winter on the respective person as “more or less likely”.

**Fig 3:**
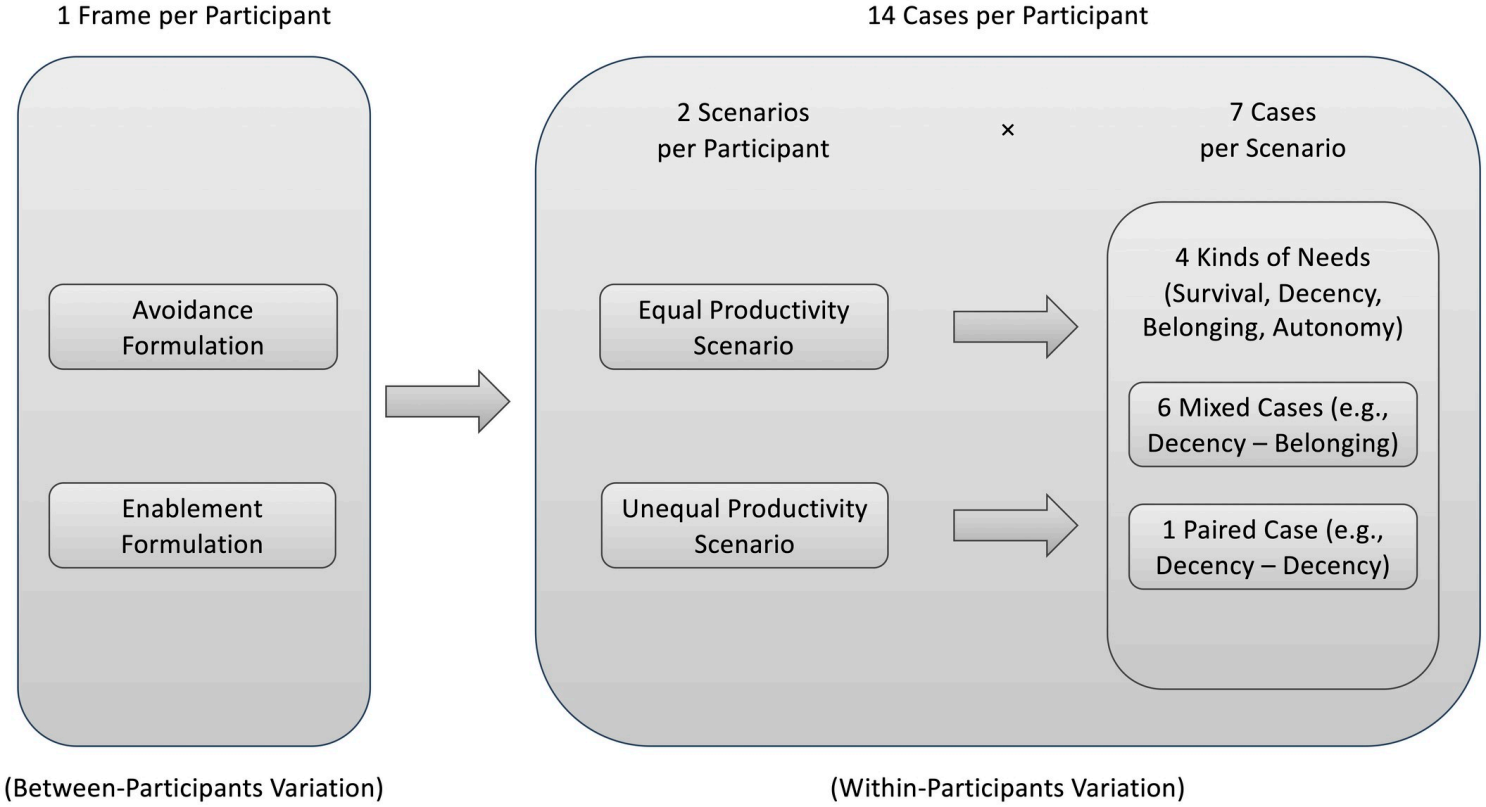
Design of Study 2.

The design of Study 2 is schematized in Fig 3. As displayed there, each participant has to make a total of 14 different allocation decisions, denoted as *Cases* in the following. For each case, they have to decide how much wood to give to Person A and Person B. Those 14 cases are split into two different *Scenarios* with 7 cases each. This within-participants variation alters whether or not Person A and B have chopped the same amount of wood. In the *Equal Productivity Scenario*, Person A and Person B have chopped 500 logs each, in the *Unequal Productivity Scenario*, Person A has only chopped 200 logs, while Person B has chopped 800 logs. Note that the total amount of logs is constant over every case and over both scenarios. We randomize whether participants started with the Equal Productivity Scenario or the Unequal Productivity Scenario. For each participant, we further randomize the order of appearance of the seven cases in each scenario.

The seven cases themselves vary what Person A and Person B need the wood for (i. e., which kind of need they experience). Here, we distinguish between *Mixed Cases* and *Paired Cases*. In Mixed Cases Person A and Person B experience two different kinds of needs, whereas in Paired Cases they experience the same kind of need. While Paired Cases give us a baseline and a consistency check, mixed cases provide us with the differences between the kinds of needs that are in our focus. Six of the seven cases a participant sees per scenario are *Mixed Cases*, containing the possible combinations that can be obtained from the four different kinds of needs without creating pairs, as depicted in Table 3. One additional case a participant sees per scenario is—randomly drawn—*one* of the four possible *Paired Cases*, which shows the same pair in both scenarios.

**Table 3:** Combinations made from kinds of needs for each case of a Scenario.

	Case					
Person	1	2	3	4	5	6
A	Survival	Survival	Survival	Decency	Decency	Belonging
B	Decency	Belonging	Autonomy	Belonging	Autonomy	Autonomy

The table shows the combinations made from the four Kinds of Needs for the six Mixed Cases of each Scenario.

Between-participants, we implement two different *Formulations* in which we present those descriptions of the different kinds of needs and their consequences either as the avoidance of negative consequences (*Avoidance Formulation*) or as the enablement of good outcomes (*Enablement Formulation*) to check whether possible effects are influenced by the way we present the kinds of needs. Half of the participants are assigned to each frame and, hence, every participant only sees one of the two frames.

Each case is presented on a separate screen (see the exemplary task in S4 Appendix for an example). On every screen, we randomize the position of Person A and Person B and, hence, the according kind of need (see Table 3), to avoid ordering effects. Participants are informed how much wood Person A and Person B have chopped each and in total on each screen. In the center of every screen, two illustrations are displayed side by side to highlight the kind of need Person A and Person B exhibit in the displayed case. Below each picture, a single sentence additionally makes clear what Person A and Person B need the wood for:

- **Survival:** “*A* [*B*] needs the wood to avoid life-threatening illness [stay healthy] in the upcoming winter.”
- **Decency:** “*A* [*B*] needs the wood to avoid feeling cold [have it warm] in the upcoming winter.”
- **Belonging:** “*A* [*B*] needs the wood in order not to be excluded from [to participate in] social life in the upcoming winter.”
- **Autonomy:** “*A* [*B*] needs the wood so that their studio does not become unusable [so that they can use their studio] in the upcoming winter.”

Note that, as in our first study, denominations (“Survival”, “Decency”, “Belonging”, and “Autonomy”) are not shown to participants. Participants are asked to enter the amount Person A and Person B should receive on a blank line in a sentence below each picture that stated “*A* [*B*] should receive ___ logs of wood”. Here, all available logs have to be distributed by them. For an exemplary task, illustrating the task screen’s structure, see S5 Appendix.

Mixed Cases, Paired Cases, and Productivity Scenarios are varied within-participants since otherwise only the treatment mean differences and not the mean values of the individual differences could be analyzed. Formulations are varied between-participants to keep the number of cases that are presented to participants manageable, hence to avoid fatigue effects, and to prevent contrast effects, which would make it difficult to find out if the wording actually makes a difference.

### Procedure

Our study, programmed in oTree [95], was conducted online in April 2021. The total sample size was *n* = 200. Participants were recruited via the private market research institute respondi, being randomly sampled from respondi’s online access panel, stratified by the characteristics gender, age, and household net income to promote external validity (see S2 Table). Sampling rates of these characteristics have been taken from the “Best for Planning” study of Germany’s *Society for Integrated Communication Research* as representative for the adult German population [98, p. 284, 291].

To control for the heterogeneity of our participants with regard to their sociodemographic backgrounds and justice attitudes, we implemented a post-experimental questionnaire, where participants were asked for their age, gender, household net income, and several justice attitudes after having completed the distribution task. Furthermore, they had to state their support for the three different distributive principles of need, equity, and equality, as well as their political orientation, all on 7-point Likert scales. Lastly, they were asked how they perceive their own sensitivity to cold (see S7 Appendix for wordings).

To facilitate internal validity and to ensure that our vignettes and instructions were read thoroughly, participants had to answer three control questions after they completed the distribution task (see S6 Appendix for wordings). The final analysis was restricted to those participants who passed at least two of the three questions. Those 200 participants were paid a flat fee of 5.40 euros, equivalent to an hourly wage of 10.80 euros. 64 participants were excluded since they failed to pass at least two of our three control questions. For them, the survey was terminated after giving two wrong answers and they were asked no further questions.

Failure rates indicate that our second question was failed more often than the first and third question (Question 1: 83 of 264 (31.44%), Question 2: 119 of 264 (45.08%), Question 3: 68 of 264 (25.76%), see S6 Appendix for the questions’ wording). Excluded participants did not diverge from the remaining participants in age, income, or gender at a significance level of 5% (Age: *χ*^2^ = 2.049, *p* = 0.727, Income: *χ*^2^ = 1.657, *p* = 0.799, Gender: *χ*^2^ = 0.047, *p* = 0.828).

### Working hypotheses

A little notation first. Let I={1,2,…,n} denote the set of participants *i* and N={(S)urvival),(D)ecency,(B)elonging,(A)utonomy)} the ordered set of the kinds of needs. Needs are ordered in terms of decreasing priority as elicited in Study 1, i. e., need *S* is greater than need *D* and so on. The Greek letters α∈N and β∈N indicate the needs of Person A and Person B, respectively. In the relative evaluation task, participant *i* is endowed with *ℓ*^*i*^ = 1000 logs of wood. They assign $0<\ell_{\alpha }^{i}\leq 1000$ logs to Person A with need *α* and $\ell_{\beta }^{i}=1000-\ell_{\alpha }^{i}$ logs to Person B with need *β*. The relative evaluation of Person A’s need *α* by participant *i* is hence given by $\Delta_{\alpha ,\beta }^{i}=\ell_{\alpha }^{i}-\ell_{\beta }^{i}=2\ell_{\alpha }^{i}-1000$.

We start with the Equal Productivity Scenario (EPS). For *paired* need evaluations *α* = *β*, we expect the mean relative need evaluation to be zero: ${\bar{\Delta }}_{\alpha ,\beta }^{\text{EPS}}=0$. For *mixed* need evaluations *α* < *β*, we expect the mean relative need evaluation to be positive, ${\bar{\Delta }}_{\alpha ,\beta }^{\text{EPS}}>0$ and to increase in the distance between absolute need evaluations. Hence, **Hypothesis 2 (Hierarchy in *Δ*)** reads as follows:

$$\begin{array}{c} {\bar{\Delta }}_{S,A}^{\text{EPS}}>{\bar{\Delta }}_{S,B}^{\text{EPS}}>{\bar{\Delta }}_{S,D}^{\text{EPS}}>0 \end{array}$$ (2)

and

$$\begin{array}{c} {\bar{\Delta }}_{D,A}^{\text{EPS}}>{\bar{\Delta }}_{D,B}^{\text{EPS}}>0\;. \end{array}$$ (3)

We further assume that participants make coherent distributive decisions. A decision is coherent if the differences we observe in mean allocations add up. In other words, it is coherent if the difference of two kinds of needs, being not next to each other in the hierarchy, equals the sum of the differences of the kinds of needs that are spanned by the original difference. Hence, **Hypothesis 3 (Coherence)** states:

$$\begin{array}{c} {\bar{\Delta }}_{S,A}^{\text{EPS}}={\bar{\Delta }}_{S,B}^{\text{EPS}}+{\bar{\Delta }}_{B,A}^{\text{EPS}}\;, \end{array}$$ (4)

$$\begin{array}{c} {\bar{\Delta }}_{S,A}^{\text{EPS}}={\bar{\Delta }}_{S,D}^{\text{EPS}}+{\bar{\Delta }}_{D,A}^{\text{EPS}}\;, \end{array}$$ (5)

$$\begin{array}{c} {\bar{\Delta }}_{S,A}^{\text{EPS}}={\bar{\Delta }}_{S,D}^{\text{EPS}}+{\bar{\Delta }}_{D,B}^{\text{EPS}}+{\bar{\Delta }}_{B,A}^{\text{EPS}}\;, \end{array}$$ (6)

and

$$\begin{array}{c} {\bar{\Delta }}_{D,A}^{\text{EPS}}={\bar{\Delta }}_{D,B}^{\text{EPS}}+{\bar{\Delta }}_{B,A}^{\text{EPS}}\;. \end{array}$$ (7)

In the Unequal Productivity Scenario (UPS), the relative need evaluation for pairs *α* = *β* does not need to be zero if productivity matters. In fact, we expect the need evaluation to be lower for the less productive Person A: ${\bar{\Delta }}_{\alpha ,\beta }^{\text{UPS}}<0$. However, we expect the impact of lower productivity on relative need evaluations to decrease with the importance of need. Hence, **Hypothesis 4 (Productivity)** reads as follows:

$$\begin{array}{c} 0>{\bar{\Delta }}_{S,S}^{\text{UPS}}>{\bar{\Delta }}_{D,D}^{\text{UPS}}>{\bar{\Delta }}_{B,B}^{\text{UPS}}>{\bar{\Delta }}_{A,A}^{\text{UPS}}\;. \end{array}$$ (8)

For mixed need evaluations, as in EPS, we expect mean relative need evaluations to increase with the distance between absolute need evaluations and we also expect relative need evaluations to be coherent.

### Results

We start by taking a look at the Paired Cases—in particular Hypothesis 4—before moving on to the Mixed Cases—in particular Hypothesis 2. After looking at the mean differences, we examine the coherence of our participants’ decisions (Hypothesis 3) and, finally, present a number of regressions as a robustness check.

#### Mean differences in allocations for Paired Cases

First, we take a look at the relative need evaluations, i. e., the way participants allocated the logs of firewood between the two persons presented to them. Since Paired Cases give us a baseline and a consistency check, we start by analyzing them. To do so, we calculate the mean of the individual differences (represented by ${\bar{\Delta }}_{\alpha ,\beta }$) of the number of logs participants gave to Person A and Person B in those four cases (see S3 Table).

**Fig 4:**
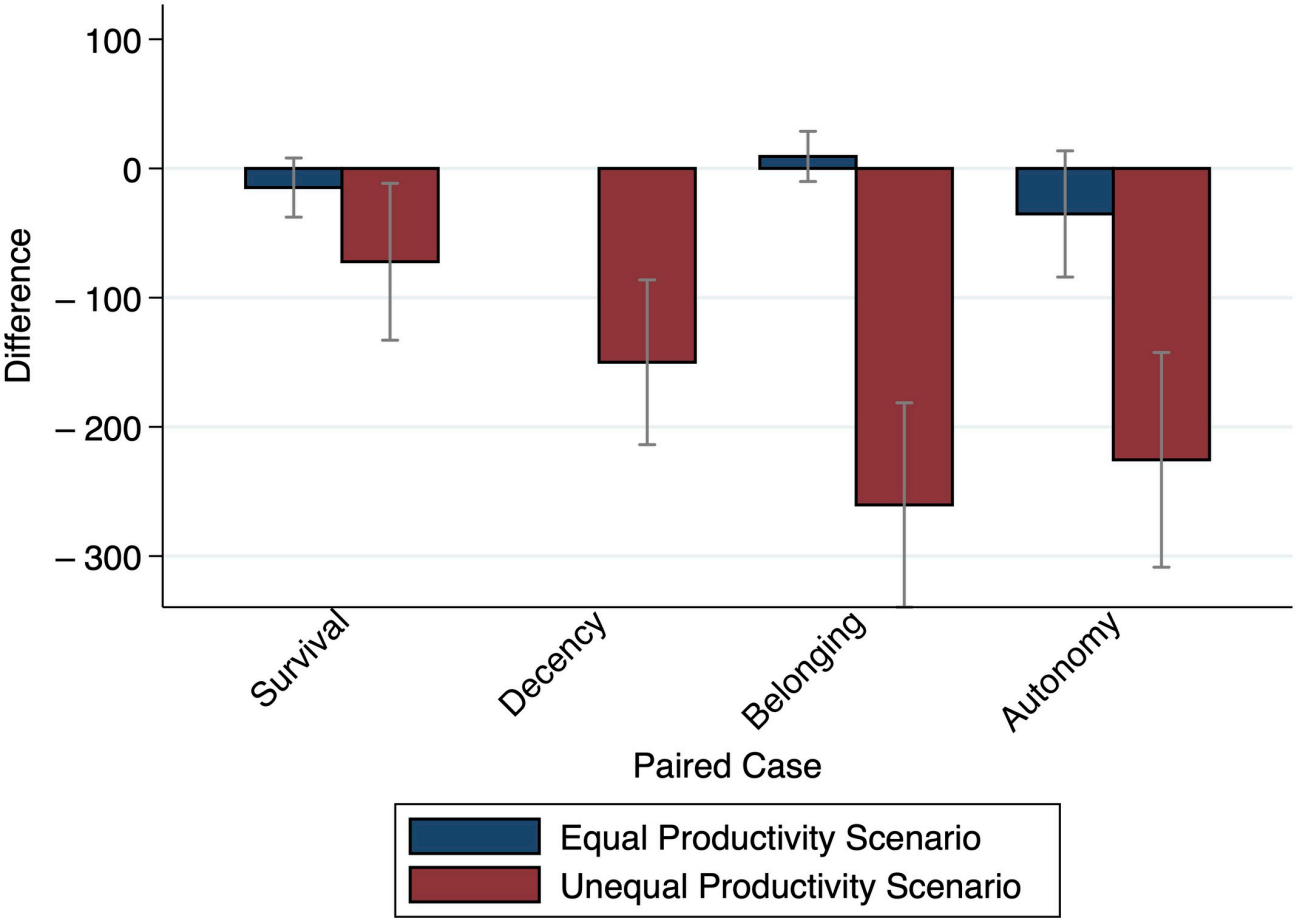
Mean differences for the 4 Paired Cases by productivity scenario. The figure shows the mean relative need evaluations ${\bar{\Delta }}_{\alpha ,\beta }$, *α* = *β*, (i. e., differences in allocations to Person A and Person B experiencing the same kind of need) by Productivity Scenario. *n* = 200.

Fig 4 shows the mean differences for the four Paired Cases. The bars are differentiated by the two Productivity Scenarios that were presented within-participants. We see that in the Equal Productivity Scenario participants by and large distribute equally between Person A and Person B, resulting in a mean difference of (roughly) around 0. Since both persons have contributed the same amount of wood and exhibit the same kind of need, this is, arguably, the only reasonable default, which is a strong indicator that our participants understood the vignette and task and took it seriously. Thus, our expectation ${\bar{\Delta }}_{\alpha ,\beta }^{\text{EPS}}=0$ is fulfilled for *α* = *β*. Moreover, two-tailed Welch tests do not reject the null hypothesis of equality of mean differences between the kinds of needs (*Survival* vs. *Decency*: *p* = 0.209, *Survival* vs. *Belonging*: *p* = 0.118, *Survival* vs. *Autonomy*: *p* = 0.459, *Decency* vs. *Belonging*: *p* = 0.352, *Decency* vs. *Autonomy*: *p* = 0.162, *Belonging* vs. *Autonomy*: *p* = 0.101).

However, in the Unequal Productivity Scenario, where Person A has cut 200 logs and Person B 800 logs, Person A receives less than Person B, which confirms our expectation ${\bar{\Delta }}_{\alpha ,\beta }^{\text{UPS}}<0$ for *α* = *β*. Interestingly, this depends on the kind of need as hypothesized above by Hypothesis 4 (Productivity). If both need the wood for *Survival*, the lower productivity of Person A has hardly any effect, that is, she gets only a little less than Person B although she has cut much less. The difference is a bit more pronounced for the second kind of need, *Decency*, and is largest for *Belonging* and *Autonomy*. But even there, Person A still gets significantly more than she has initially contributed. Two-tailed Welch tests confirm these observations (*Survival* vs. *Decency*: *p* = 0.085, *Survival* vs. *Belonging*: *p* ≤ 0.001, *Survival* vs. *Autonomy*: *p* = 0.004, *Decency* vs. *Belonging*: *p* = 0.035, *Decency* vs. *Autonomy*: *p* = 0.160, *Belonging* vs. *Autonomy*: *p* = 0.551).

In S4 Table, we additionally report means of absolute percentage deviations of the share that Person A receives from the share that Person A herself contributed. This deviation can be interpreted as a kind of elasticity of need satisfaction for productivity; the larger this deviation, the more important needs are considered. In the Equal Productivity Scenario, as would be expected, this fluctuates around roughly 1%. In the Unequal Productivity Scenario, on the other hand, we get a benchmark for the marginal effect of productivity for the same kinds of needs. This is a first measure of the importance of the different kinds of needs. The more productivity matters, the less the equality of needs matters. The difference is highest for *Survival* (10.556%), followed by *Decency* (9%), *Autonomy* (7.486%), and *Belonging* (6.791%).

#### Mean differences in allocations for Mixed Cases

Next, we consider the Mixed Cases. Again, we calculate the mean relative need evaluation in terms of the individual differences of the number of logs participants gave to Person A, experiencing one kind of need, and Person B, this time experiencing another, less basic, kind of need (see S3 Table).

**Fig 5:**
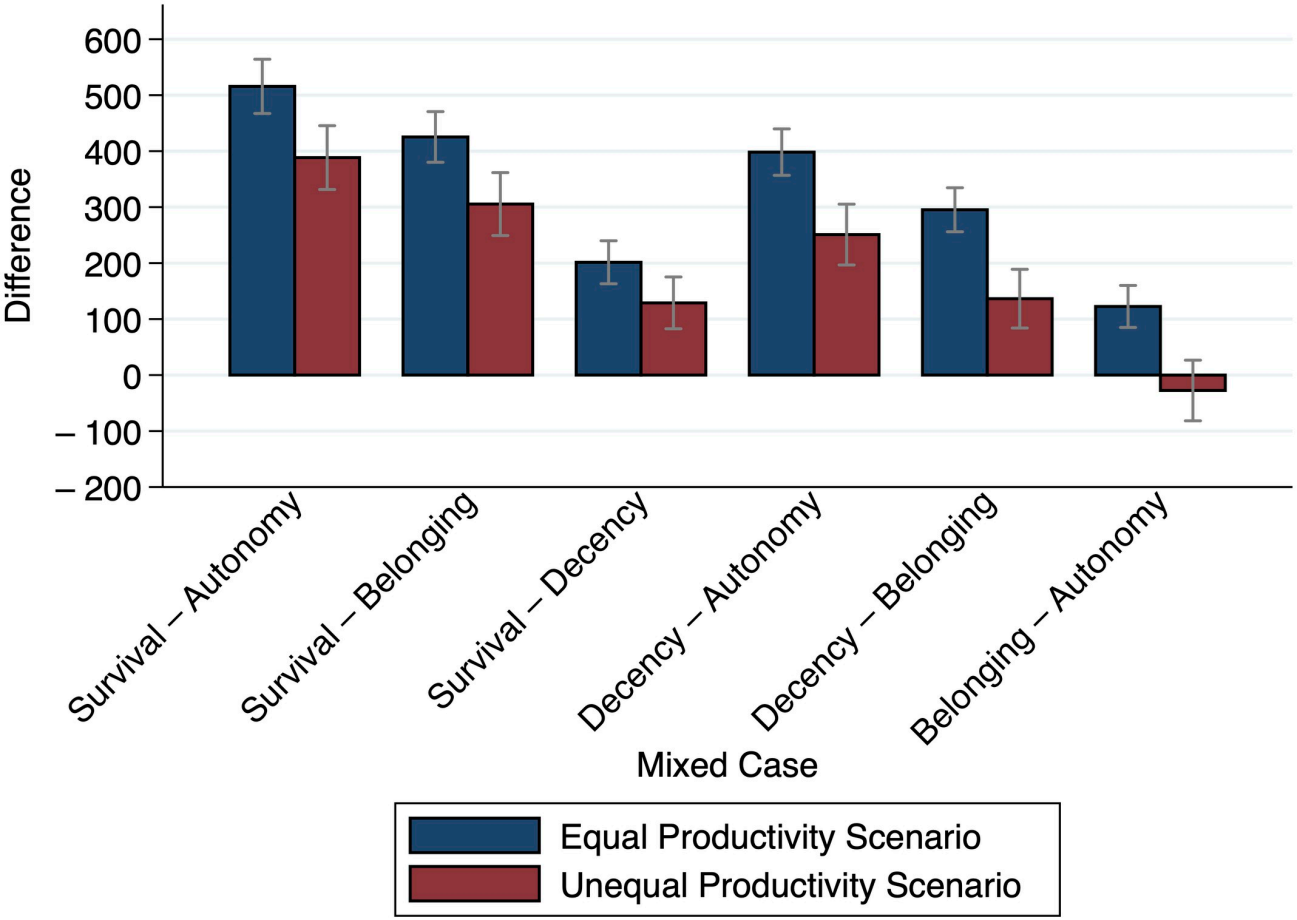
Mean differences for the 6 Mixed Cases by productivity scenario. The figure shows the mean relative need evaluations ${\bar{\Delta }}_{\alpha ,\beta }$, *α* < *β*, (i. e., differences in allocations to Person A and Person B, experiencing different kinds of needs) by Productivity Scenario. *n* = 200.

Fig 5 presents the mean relative need evaluation for the six possible Mixed Cases that can be made with Person A and Person B experiencing different kinds of needs, again by Productivity Scenario. As suspected, in both Productivity Scenarios, the relative need evaluations are positive (except for *Belonging—Autonomy* in the Unequal Productivity Scenario) and furthermore, it becomes apparent that in the Unequal Productivity Scenario the mean evaluations are smaller than in the Equal Productivity Scenario for every combination. In S3 Table, we report the respective two-tailed Welch’s t-tests for the difference between the Productivity Scenarios confirming that relative need evaluations for all cases are significantly lower in the Unequal Productivity Scenario.

We see, as postulated by Hypothesis 2 (Hierarchy), that the mean relative need evaluation increases with the distance between the absolute need evaluations. If Person A needs *Survival* and Person B “only” needs *Autonomy*, the relative need evaluation (combined over EPS and UPS) is highest and greater than if B has a *Belonging* need (*p* = 0.008) or *Decency* need (*p* ≤ 0.001), and the relative need evaluation *Survival—Decency* is also greater than *Survival—Belonging* (*p* ≤ 0.001, two-tailed Welch t-tests). Analogously, if Person A has a *Decency* need, the relative need evaluation of *Decency—Autonomy* is greater than *Decency—Belonging* (*p* = 0.001, two tailed Welch t-test). Thus, consistent with the literature, it can be concluded that there is a clear hierarchy of the four kinds of needs in which *Survival* comes before *Decency*, *Decency* comes before *Belonging*, and *Belonging* comes before *Autonomy*.

#### Summation of allocations

In addition to the hierarchy observed above, we assume that rational people make coherent relative need evaluations when distributing resources among people who experience different kinds of needs. As has been noted in connection with Hypothesis 3 (Coherence), we speak of coherent relative need evaluations when they add up. This is given if the relative need evaluation (Δ_*α*,*β*_) of two kinds of needs, being not next to each other in the hierarchy, equals the sum of the relative need evaluations that are spanned by the original relative need evaluation.

**Fig 6:**
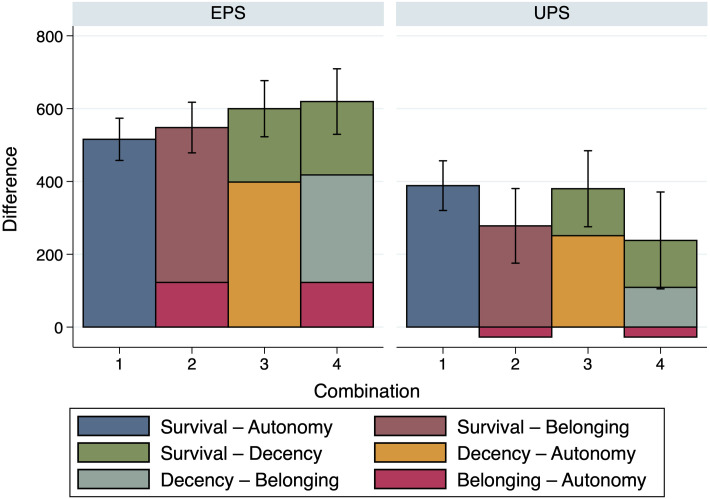
Comparison of importance ratings: Survival—Autonomy. The figure shows a comparison between the reference case *Survival—Autonomy* and the possible additions by Productivity Scenario. *n* = 200.

**Fig 7:**
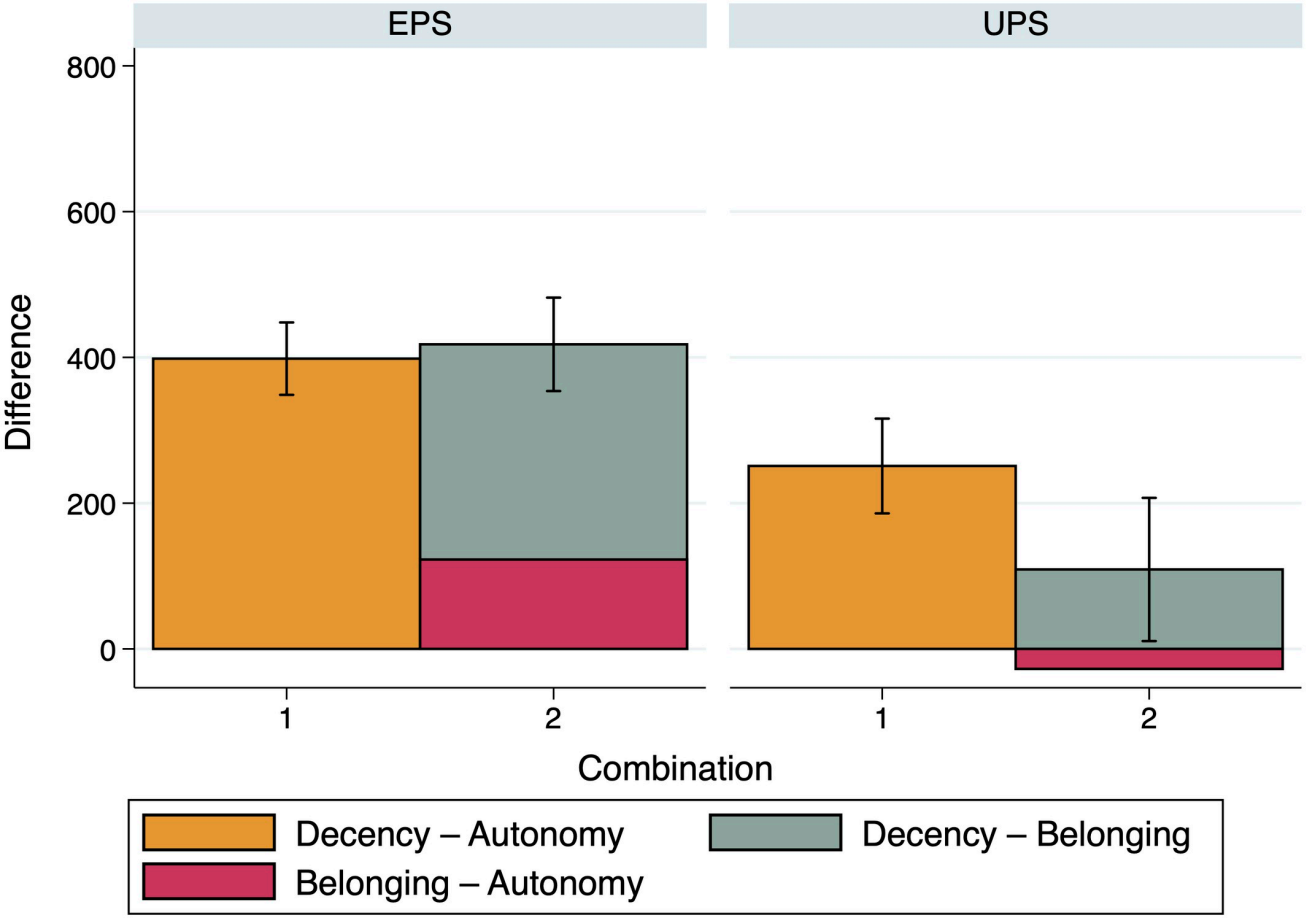
Comparison of importance ratings: Decency—Autonomy. The figure shows a comparison between the reference case *Decency—Autonomy* and the possible addition by Productivity Scenario. *n* = 200.

**Fig 8:**
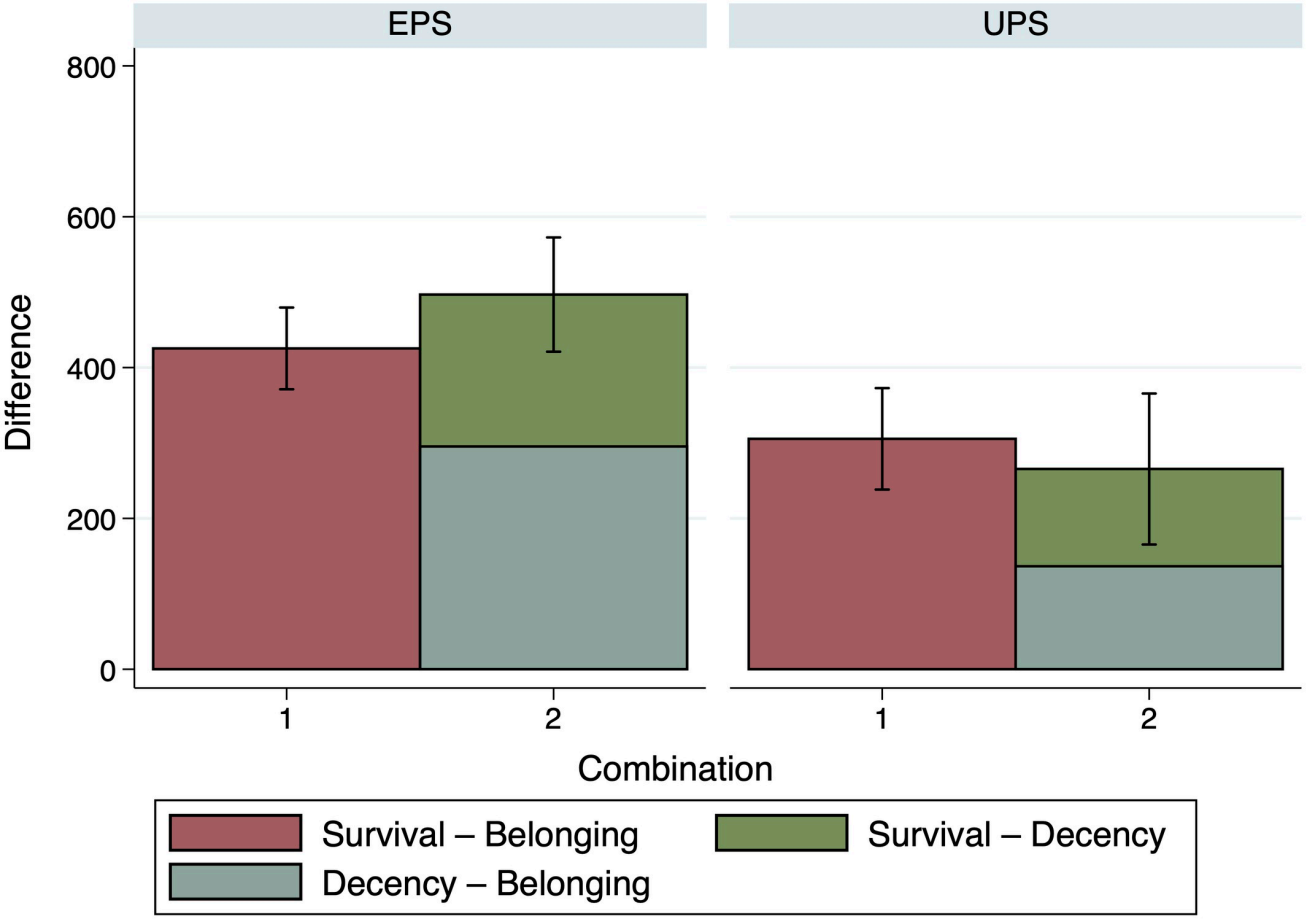
Comparison of importance ratings: Survival—Belonging. The figure shows a comparison between the reference case *Survival—Belonging* and the possible addition by Productivity Scenario. *n* = 200.

Figs 6–8 indicate that this, indeed, seems to be the case. In Fig 6, the first bar shows the difference of the case *Survival—Autonomy* as reference, both in the Equal and the Unequal Productivity Scenario. The following three bars then show the three possible additions, as introduced in Eqs (3) and (5), above. In Fig 7, the first bar shows the case *Decency—Autonomy* as reference, again both for the Equal and the Unequal Productivity Scenario, followed by the analogous addition of *Belonging—Autonomy* and *Decency—Belonging* in the next bar. In Fig 8, the first bar shows the case of *Survival—Belonging*, once more for both the Equal and the Unequal Productivity Scenario, followed by the analogous addition of *Decency—Belonging* and *Survival—Decency* in the second bar.

To assess whether or not the references and the additions differ significantly from each other in the sum of relative need evaluations, we ran a number of one-way ANOVAs with Bonferroni correction; one for every Productivity Scenario regarding the combinations shown in Fig 6 (Equal Productivity Scenario: *F*(3, 796) = 2.24, *p* = 0.083, Unequal Productivity Scenario: *F*(3, 796) = 2.82, *p* = 0.038) as well as regarding the combinations shown in Fig 7 (Equal Productivity Scenario: *F*(1, 398) = 0.320, *p* = 0.571, Unequal Productivity Scenario: *F*(1, 398) = 8.010, *p* = 0.005) and Fig 8 (Equal Productivity Scenario: *F*(1, 398) = 3.240, *p* = 0.073, Unequal Productivity Scenario: *F*(1, 398) = 0.610, *p* = 0.437). The ANOVAs indicate that the additions have total values that do not differ significantly from the reference values, except for the addition of *Decency—Belonging* and *Belonging—Autonomy* compared to the reference of *Decency—Autonomy* in the Unequal Productivity Scenario (with *p* = 0.005, see Fig 7).

Although most of the differences between the combinations of need comparisons are insignificant and, therefore, coherence cannot be rejected, a certain response pattern seems to emerge. In the Equal Productivity Scenario, some combined evaluations exceed the reference case, which increases with the number of comparisons combined (see, in particular, the left panel of Fig 6). Analogously, in the Unequal Productivity Scenario, some combined evaluations fall short of the reference case, which also seems to increase when multiple comparisons are combined (see, in particular, the right panel of Fig 6). Thus it seems possible that the participants, in the scenario where Persons A and B exhibit the same productivity but different needs, basically grant the person with greater need a kind of “bonus” that *accumulates* when several comparisons are combined. The Unequal Productivity Scenario creates a situation where (too much) distribution towards the person with greater need is itself perceived as unjust in terms of equity and, therefore, the person with greater need receives a “malus” that also accumulates when several comparisons are combined.

An obvious explanation for this decision pattern would be a gain-loss domain effect, such as that postulated by Prospect Theory (see, e. g., [73, 99]). That reference points and loss aversion play a role in distributional decisions of “social planners” has been shown by a number of experimental studies (e. g., [100]). Bauer and colleagues have shown that high accountability for a lack of resources gives rise to an asymmetry where a disadvantaged person’s compensation is significantly smaller when her disadvantage is due to lower productivity instead of greater need (see [19]). Moreover, it has recently been shown in a vignette experiment that need is perceived as a reference point below which the aggregate justice evaluation function is convex and above which the justice evaluation is concave (see [101]).

#### Regressions

Finally, as a robustness check, we turn to a number of Tobit panel regressions, reported in Table 4 using the participants’ ID as the panel variable and the case number as the time variable (in all models considered, a likelihood-ratio test rejects the null hypothesis that the pooled estimator performs as well as the panel model). We focus only on Mixed Cases, since we are mostly interested in Hypothesis 2 (Hierarchy). We chose to use Tobit models since our dependent variable—the relative need evaluation for Mixed Cases, i. e., the difference between the logs distributed to Persons A with need *α* and Person B with need *β*, *α* < *β*—contains two left censored and 80 right censored observations. In all regressions, the mixed case with the largest relative need evaluation (*Survival—Autonomy*) serves as our reference category. In Model (I), we estimate the relative need evaluations for the six Mixed Cases. In Model (II), we interact them with the Productivity Scenario (with Equal Productivity as the reference category). The influence of control variables such as Age, Gender, and Household Net Income on the relative need evaluation is examined in Model (III). These covariates are reported in S7 Table. In Model (IV), we interact the Mixed Cases with the Formulation (with Avoidance as the reference category). Since the Formulation has no significant effect on the relative need evaluation in Model (IV) and does not improve the model fit in terms of the log likelihood, we omit estimating a fully interacted model. Note that the margins (i. e., the predicted means of the relative need evaluations for the Mixed Cases) and tests can be taken from S5 Table for Model (I) and S6 Table for Models (II)–(IV).

**Table 4:** Allocation difference between Person A and B: Regression results.

Coefficient	(I)	(II)	(III)	(IV)
Survival—Belonging	-92.99 ***	-99.34 ***	-106.17 ***	-104.4 ***
	(19.57)	(26.79)	(26.77)	(28.58)
Survival—Decency	-298.1 ***	-329.2 ***	-337.9 ***	-299.3 ***
	(19.53)	(26.73)	(26.71)	(28.51)
Decency—Autonomy	-134.3 ***	-127.3 ***	-133.6 ***	-139.5 ***
	(19.56)	(26.78)	(26.76)	(28.56)
Decency—Belonging	-246.4 ***	-233.7 ***	-249.3 ***	-270.6 ***
	(19.54)	(26.74)	(26.72)	(28.52)
Belonging—Autonomy	-416.2 ***	-408.6 ***	-410.9 ***	-411.6 ***
	(19.53)	(26.72)	(26.70)	(28.50)
Unequal Productivity (Benchmark: Equal)		-134.8 ***	-150.0 ***	
		(26.80)	(28.59)	
Survival—Belonging × Unequal Productivity		12.77	15.31	
		(37.81)	(40.32)	
Survival—Decency × Unequal Productivity		62.50 *	73.11 *	
		(37.73)	(40.23)	
Decency—Autonomy × Unequal Productivity		-13.98	-10.56	
		(37.79)	(40.30)	
Decency—Belonging × Unequal Productivity		-25.19	-23.91	
		(37.75)	(40.25)	
Belonging—Autonomy × Unequal Productivity		-15.01	-8.33	
		(37.72)	(40.22)	
Enablement (Benchmark: Avoidance)				-32.01
				(43.68)
Survival—Belonging × Enablement				22.85
				(39.11)
Survival—Decency × Enablement				2.480
				(39.04)
Decency—Autonomy × Enablement				10.49
				(39.10)
Decency—Belonging × Enablement				48.44
				(39.05)
Belonging—Autonomy × Enablement				-9.123
				(39.03)
Constant	465.1 ***	532.3 ***	765.9 ***	481.1 ***
	(21.86)	(25.46)	(121.44)	(30.88)
Control variables	*No*	*No*	*Yes*	*No*
*n*	2400	2400	2196	2400
log likelihood	-16624	-16550	-15101	-16622
Wald *χ*^2^	600.020 ***	794.280 ***	826.36 ***	603.90 ***
*σ*_*u*_	238.8 ***	239.4 ***	188.5 ***	238.6 ***
	(13.33)	(13.27)	(11.64)	(13.32)
*σ*_*e*_	274.7 ***	265.3 ***	270.4 ***	274.5 ***
	(4.238)	(4.094)	(4.369)	(4.235)
LR *χ*^2^(*σ*_*u*_ = 0)	876.87 ***	941.10 ***	508.47 ***	876.61 ***

The table reports the results of a Tobit random-effects panel regression. Endogenous variable: relative evaluation of Person A’s need (Δ_*α*,*β*_). Reference group: Survival—Autonomy. First row: coefficients, second row: standard errors in parentheses. Control variables include Age, Gender, and Household Net Income (see S7 Table). 2 (80) left-censored (right-censored) observations. Significance levels:

* *p* < 0.10,

** *p* < 0.05,

*** *p* < 0.01.

Model (I) shows that the mean relative need evaluation of the reference case *Survival—Autonomy* (i. e., the regression constant) is 465.1. This means that Person A with a *Survival* need receives approximately 465 more logs than Person B with a need for *Autonomy*. The estimation coefficients for the other five mixed cases are all significantly negative, i. e., the relative need evaluation of Person A is lower. The corresponding margins can be found in S5 Table. The table shows that, as hypothesized, the relative need evaluations of all Mixed Cases are significantly different from zero. Wald tests confirm what we have already seen in Fig 4, namely that the relative need evaluation increases with the difference of the absolute need evaluations, which creates a hierarchy of need evaluations (Hypothesis 2).

Model (II) shows that interacting the Mixed Cases with the Productivity Scenario improves the fit of the regression in terms of the log likelihood. Person A’s lower productivity has a significant negative impact on her relative need evaluation of about 135 logs. However, except for *Survival—Decency*, the interaction terms are insignificant, i. e., the negative influence of lower productivity applies almost equally to all Mixed Cases. Hence, in contrast to the Paired Cases, there is no significant interaction between the absolute importance of Person A’s needs and the negative effect of her lower productivity (Hypothesis 4). The upper panel of S6 Table contains the margins for both Productivity Scenarios. As in Model (I), all relative need evaluations are significantly positive (except for *Belonging—Autonomy* in the Unequal Productivity Scenario); they are significantly larger in the Equal Productivity Scenario than in the Unequal Productivity Scenario; and they exhibit a clear hierarchy (Hypothesis 2).

In Model (III), we add household net income, gender, and age, as well as political attitude and the importance of productivity, equality, and need, for the participants’ decisions as control variables (these covariates are reported in S7 Table). Of the covariates, only the questions for the importance of need, productivity, and equality as decision criteria are significant. Here, stronger emphasis on the importance of productivity leads to a smaller difference between the logs distributed to Persons A and B with a regression coefficient of −78.36, so does—to a lesser extent—an emphasis on the importance of equality with a coefficient of −17.282. Importance of need, then, works in the opposite direction, leading to a larger difference with a coefficient of 23.07. As can be taken from the middle panel of S6 Table, all results regarding our hypotheses are robust to the inclusion of the covariates.

As noted above, Model (IV), then, shows that the two Formulations—one of them in terms of avoidance of harm, the other one in terms of enablement of something good—make no overall difference with respect to the relative need evaluations (the constant term for Enablement and the interactions with the Mixed Cases are insignificant) and barely improve the fit of the regression as compared to Model (I). In the fully interacted model, which is not included here due to lack of space, only the interaction term *Enablement × Unequal Productivity* is weakly significant (*p* = 0.079) and positive. Hence, the needier and less productive Person A tends to be granted slightly more logs in the Enablement Formulation than in the Avoidance Formulation, i. e., the lower productivity of Person A is somewhat less important. Overall, however, it can be said that the verbal framing of the decision task had almost no effect on the relative need evaluations, and it can be assumed that the neutral graphic representation of the four kinds of needs dominated the subjects’ need evaluations.

Almost identical results are obtained for the regressions reported in Table 4 when only including participants who answered all control questions correctly (*n* = 116); in Models (II) and (III), the interaction *Survival—Decency × Unequal Productivity* becomes insignificant, while the interaction *Decency—Belonging × Unequal Productivity* becomes weakly significant (*p* < 0.10).

## Conclusion

In this paper, we presented the results of two vignette studies with online samples of the German adult population, investigating how laypeople evaluate four different kinds of needs, namely, survival, decency, belonging, and autonomy. Participants of both studies were recruited via the online platform respondi. Samples were stratified by gender, age, and income.

In the first study, participants (*n* = 100) had to evaluate the importance of the different kinds of needs in absolute terms on a 7-point Likert scale. To this end, they were first presented with vignettes in which hypothetical persons experienced the different kinds of needs. Each vignette was accompanied by an illustration. We hypothesized that the four kinds of needs would not be perceived as equally important, but that there would be a hierarchy. This hypothesis receives very clear support from the data; survival is rated highest, decency comes second, followed by belonging and autonomy.

In the second study, participants (*n* = 200) had to make distributive decisions. They were presented with a series of cases in which two hypothetical persons experienced (mostly) different kinds of needs. They then had to decide how to divide a scarce amount of a good between the two. That is, they had to trade-off the satisfaction of one kind of need with another kind of need. Within-participants, we have also varied whether the two persons contributed equally or differently to the amount available for (re)distribution. Between-participants, we have further varied whether the kinds of needs were presented in an avoidance or enablement formulation. The results lend further support to the hierarchy found in Study 1. Additionally, we can see that the productivity of the two hypothetical persons has an influence on how participants distribute. If one person has contributed less to the available amount, they tend to receive less of it. In addition, we were able to verify that the distribution decisions of our participants are internally coherent insofar as they add up. Moreover, the type of formulation had no influence on the distribution of our participants, which shows that the effects found do not depend on minor differences in the wording.

Our results fit well with the hierarchizations from psychological and philosophical literature. There are, of course, differences in the details, but the general orientation is quite similar. When Alderfer proposes the triptych of existence needs, relatedness needs, and growth needs, or when Maslow suggests physiological needs, safety needs, social needs, esteem needs, and self-actualization needs, this comes fairly close to the ordering we found for our survival needs, decency needs, belonging needs, and autonomy needs. Moreover, this ordering echoes the widespread idea from the philosophical literature that there are basic needs beyond the physiological minimum that must not be ignored just because survival comes first. These needs are taken to be directed at social participation and human development. Lastly, our results have interesting implications for the role of both needs and different justice principles in the context of distributive and social policy. Our results show that, even in the case of the most basic needs, productivity still plays a role in distributive decisions. Differentiating both with regard to the kinds of needs and to the contribution (and further factors of accountability, see [19]) might be worthwhile in researching the relation of perceived deservingness and policy support (see, e. g., [102, 103]).

## Supporting information

S1 Appendix Instructions of Study 1. (ZIP)

S2 Appendix Control questions of Study 1. (ZIP)

S3 Appendix Additional questions of Study 1. (ZIP)

S4 Appendix Instructions of Study 2. (ZIP)

S5 Appendix Exemplary task of Study 2. (ZIP)

S6 Appendix Control questions of Study 2. (ZIP)

S7 Appendix Additional questions of Study 2. (ZIP)

S1 Table Sample of Study 1 by gender, age, and income. (ZIP)

S2 Table Sample of Study 2 by gender, age, and income. (ZIP)

S3 Table Mean differences for cases by productivity scenario. (ZIP)

S4 Table Percentage deviation of the share that Person A receives from the share that Person A contributed for Paired cases by productivity scenario. (ZIP)

S5 Table Margins (Relative need evaluations) of Model (I). (ZIP)

S6 Table Margins (Relative need evaluations) by productivity scenario and frame. (ZIP)

S7 Table Control variables for Study 2. (ZIP)
