# Supplementary material for: Winter is coming: How laypeople think about different kinds of needs
Source: PLoS One. 2023 Nov 27;18(11):e0294572. doi: 10.1371/journal.pone.0294572 (PMC10681262; doi:10.1371/journal.pone.0294572)
Supplement: S1 Appendix — (ZIP) [file pone.0294572.s001.zip › S1_Appendix.pdf]

S1 Appendix Instructions of Study 1

Welcome screen In this survey, we are interested in your personal opinion and judgment. Therefore, there are no correct or incorrect answers in this study. Taking part in this study is voluntary, and you can drop out at any time.

You will probably need about 15 minutes if you work intently. It is important that you complete the study without interruption and without closing your browser. If you cannot avoid closing your browser, you can continue the study by clicking on the link in the invitation at Mingle again.

In the course of the study, we will give you a total of three attention checks. With these questions we want to make sure that you read and understand the instructions correctly. If you answer more than one of these questions incorrectly, you will automatically be excluded from the study.

We will analyze your answers together with the answers of all other participants in this study. All data will be stored in an anonymous format so that no participant can be identified. The results of the study will be published. They may influence future research and may be used to inform policymakers.

Thank you for participation!

Vignette text *Note: We randomized the names displayed, denoted as A, B, C, and D below ("Bauer", "Becker", "Fischer", "Hoffmann", "Klein", "Koch", "Meyer", "Müller", "Neumann", "Richter", "Schäfer", "Schmidt", "Schneider", "Schröder", "Schulz", "Schwarz", "Wagner", "Weber", "Wolf", and "Zimmermann", based on frequent German surnames), as well as the order the four kinds of needs appeared in. Next to each kind of need, a thumbnail of the picture illustrating this kind of need was displayed.*

Please imagine four people with the names A, B, C, and D. All are in need for wood. They need the wood for different reasons. On this page, we present to you the different reasons for which A, B, C, and D need the wood. On the following pages, you will be asked how important it is that the respective person's need is met.

A: A needs the wood to make sure to survive the coming winter. If A receives less than he needs, it will be so cold in his hut that he is very likely to become life-threateningly ill. The less wood he receives, the higher the probability that he will become life-threateningly ill.

B: B needs the wood in order not to freeze in the coming winter. The members of the community to which B belongs agree that one cannot live in dignity if one has to freeze. If B receives less than he needs, it becomes unacceptably cold in his hut. The less wood he receives, the more often he will freeze.

C: C needs the wood to be able to participate regularly in the social life of his community in the coming winter. It is common practice to meet at the community center and everyone brings wood with which to heat it. If C receives less than he needs, he will not be able to participate regularly in the social life. The less wood he receives, the less often he will be able to come to meetings at the community center.

D: D needs the wood to be able to use his studio regularly in the coming winter. He creates art there in his spare time. If D receives less than he needs, he will not be able to use his studio regularly. The less wood he receives, the less often he will be able to create art in his studio.

Task *Note: The needs of Person A, B, C, and D were displayed on separate screens. Their names were identical to those from the instructions screen. The order of the four screens was randomized. On each screen, we displayed a picture illustrating the kind of need in question. Bellow each picture, a single sentence summed up the kind of need. Participants had to enter their rating on a scale from 1 to 7. An additional option for "no answer/I don't know" was included.*

Please indicate how important you think the following kind of need is that A [B, C, D] can meet by using firewood.

A: A needs the wood to avoid becoming life-threateningly ill.

B: B needs the wood to avoid freezing.

C: C needs the wood to participate in social life.

D: D needs the wood to use his studio.

How much does A [B, C, D] need the wood in this case? Please give your answer on the following scale from 1 ("Does not need the wood at all") to 7 ("Does absolutely need the wood").
