# Supplementary material for: Winter is coming: How laypeople think about different kinds of needs
Source: PLoS One. 2023 Nov 27;18(11):e0294572. doi: 10.1371/journal.pone.0294572 (PMC10681262; doi:10.1371/journal.pone.0294572)
Supplement: S2 Appendix — (ZIP) [file pone.0294572.s002.zip › S2_Appendix.pdf]

S2 Appendix Control questions of Study 1

Note: Options for Questions 2 and 3 were displayed in randomized order.

Question 1: Please describe how often you reflect on justice issues in your daily life and what this means to you.

We ask this question to ensure that the tasks are read carefully. If you are reading this, please enter the number 42 in the field below instead of an answer to the question itself.

Have you ever reflected on justice issues?

Question 2: How many reasons to need wood have we presented to you?

- ☐ 3
- ☐ 4
- ☐ 5

Question 3: Which statements apply to this study? Multiple answers are possible.

- ☐ Wood is needed to renovate a building.
- ☐ Wood is needed to carve together.
- ☐ Wood is needed to heat.
