# Supplementary material for: Winter is coming: How laypeople think about different kinds of needs
Source: PLoS One. 2023 Nov 27;18(11):e0294572. doi: 10.1371/journal.pone.0294572 (PMC10681262; doi:10.1371/journal.pone.0294572)
Supplement: S3 Appendix — (ZIP) [file pone.0294572.s003.zip › S3_Appendix.pdf]

S3 Appendix Additional questions of Study 1

Note: An additional option for "no answer/I don't know" was included.

Political orientation In politics, one speaks of left-wing and right-wing. How would you describe your own political position in general? Where on a scale of 1 (left) to 7 (right) would you place yourself?

Sensitivity to cold On a scale from 1 (not at all sensitive to cold) to 7 (very sensitive to cold), how sensitive are you to cold?
