# Supplementary material for: Winter is coming: How laypeople think about different kinds of needs
Source: PLoS One. 2023 Nov 27;18(11):e0294572. doi: 10.1371/journal.pone.0294572 (PMC10681262; doi:10.1371/journal.pone.0294572)
Supplement: S4 Appendix — (ZIP) [file pone.0294572.s004.zip › S4_Appendix.pdf]

S4 Appendix   Instructions of Study 2

Welcome screen   In this survey, we are interested in your personal opinion and judgment. Therefore, there are no correct or incorrect answers in this study. Taking part in this study is voluntary, and you can drop out at any time.

You will probably need about 30 minutes if you work intently. It is important that you complete the study without interruption and without closing your browser. If you cannot avoid closing your browser, you can continue the study by clicking on the link in the invitation at Mingle again.

In the course of the study, we will give you a total of three attention checks. With these questions we want to make sure that you read and understand the instructions correctly. If you answer more than one of these questions incorrectly, you will automatically be excluded from the study.

We will analyze your answers together with the answers of all other participants in this study. All data will be stored in an anonymous format so that no participant can be identified. The results of the study will be published. They may influence future research and may be used to inform policymakers.

Thank you for participation!

Instructions   Your task will be to distribute firewood between two people. We will present you with a number of different scenarios and ask you to imagine that they are real. Please take the time to put yourself in the position of the scenarios and come to a personal judgment.

Vignette text   *Note: The four reasons were displayed to participants either in the Avoidance or in the Enablement Formulation. We randomized the names displayed, denoted as A and B below ("Bauer", "Müller", "Schmidt", "Schneider", "Fischer", "Weber", and "Meyer", based on frequent German surnames), as well as the position of the two persons (randomizing whether the more productive or the more needy person appears on the left side of the screen).*

Please imagine two people with the names A and B. A and B do not know each other. Both are in need of wood. The community of A and B allows them to chop wood in the community forest for a certain period of time. Both have little money and therefore have no other way to get wood.

On the coming pages, we will present you with a total of 14 cases where A and B need the wood for different reasons. On each page, we will tell you what A needs the wood for and what B needs the wood for. You will then be asked to divide the wood as fairly as possible between A and B.

Please note that you have to make the following trade-off: The more wood you give to one person, the less you can give to the other. It is not possible to completely meet the needs of both people at the same time. In each of the 14 cases, the available amount of wood will only be enough to completely cover the needs of one of the two people; the other person would then go away empty-handed.

We now present to you the four different reasons for which A and B may need the wood. These four reasons have to do with the coming winter. Since you need to distribute the wood in advance without knowing exactly how cold the winter will be, we describe the expected effects of the winter on the people as more or less likely.

Please read the descriptions of the four reasons carefully.

Reason 1 (Avoidance Formulation): The person needs the wood to avoid falling life-threateningly ill and dying from it in winter. They heat their hut exclusively with wood. The more logs the person gets, the less likely they are to fall life-threateningly ill.

If the person gets no wood at all, they will certainly fall life-threateningly ill. If the person gets all the available wood, they will certainly not fall life-threateningly ill.

Reason 1 (Enablement Formulation): The person needs the wood to stay healthy and survive in winter. She heats her hut exclusively with wood. The more logs the person gets, the higher the probability that they will stay healthy. If the person gets no wood at all, they will certainly not stay healthy. If the person gets all the available wood, they will certainly stay healthy.

Reason 2 (Avoidance Formulation): The person needs the wood to not freeze in winter. They heat their hut exclusively with wood. The more logs the person gets, the less likely they will freeze. If the person gets no wood at all, they will certainly freeze. If the person gets all the wood available, they will certainly not freeze.

Reason 2 (Enablement Formulation): The person needs the wood to be warm in winter. They heat their hut exclusively with wood. The more logs the person gets, the higher the probability that they will be warm. If the person does not get any wood at all, they will certainly not have it warm. If the person gets all the available wood, they will certainly have it warm.

Reason 3 (Avoidance Formulation): The person needs the wood in order not to be excluded from social life in winter, since it is common practice to meet in the community center and everyone brings wood with which to heat it. The more logs the person gets, the less likely they are to be excluded from social life. If the person gets no wood at all, they will certainly be excluded from social life. If the person gets all the available wood, they will certainly not be excluded from social life.

Reason 3 (Enablement Formulation): The person needs the wood to participate in social life in winter, since it is common practice to meet in the community center and everyone brings wood to heat it. The more logs the person gets, the more likely they are to participate in social life. If the person does not get any wood at all, they will certainly not participate in social life. If the person gets all the available wood, they will certainly participate in social life.

Reason 4 (Avoidance Formulation): The person needs the wood so that their studio does not become unusable in winter. They heat their studio exclusively with wood. There, they create art in their free time. The more logs the person gets, the less likely it is that their studio will become unusable. If the person gets no wood at all, their studio will certainly be unusable. If the person gets all the available wood, their studio will certainly not be unusable.

Reason 4 (Enablement Formulation): The person needs the wood so that they can use their studio in the winter. They heat their studio exclusively with wood. There, they create art in their free time. The more logs the person gets, the more likely they are to use their studio. If the person gets no wood at all, they will certainly not use their studio. If the person gets all the available wood, they will certainly use their studio.

Scenario introduction *Note: Before each scenario, a single sentence was displayed to introduce participants to the productivity of Person A and B in the next seven cases.*

Equal Productivity Scenario: In the following 7 cases that we show you, A and B have each cut 500 logs of wood.

Unequal Productivity Scenario: In the following 7 cases that we show you, A has cut 200 and B 800 logs of wood.
