# Supplementary material for: Winter is coming: How laypeople think about different kinds of needs
Source: PLoS One. 2023 Nov 27;18(11):e0294572. doi: 10.1371/journal.pone.0294572 (PMC10681262; doi:10.1371/journal.pone.0294572)
Supplement: S5 Appendix — (ZIP) [file pone.0294572.s005.zip › S5_Appendix.pdf]

S5 Appendix Exemplary Task of Study 2

A and B have cut 500 logs each [A has cut 200 and B has cut 800 logs]. So both persons have cut a total of 1,000 logs. In the empty spaces below, please make the distribution to both people that you think is most just.

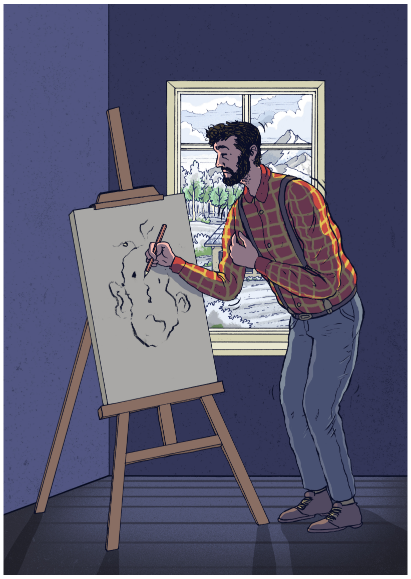

A needs the wood so that their studio does not become unusable in the winter.

A should receive ___ logs of wood.

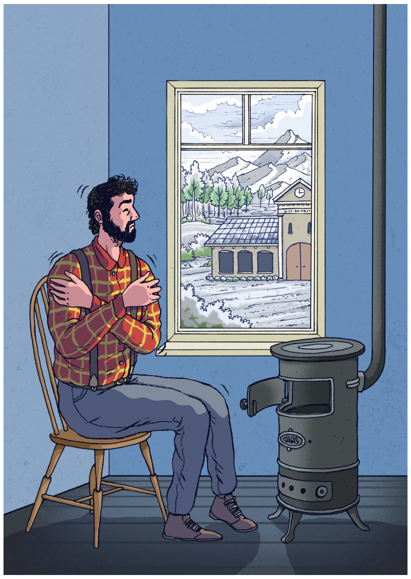

B needs the wood to avoid freezing in the winter.

B should receive ___ logs of wood.
