# Supplementary material for: Winter is coming: How laypeople think about different kinds of needs
Source: PLoS One. 2023 Nov 27;18(11):e0294572. doi: 10.1371/journal.pone.0294572 (PMC10681262; doi:10.1371/journal.pone.0294572)
Supplement: S7 Appendix — (ZIP) [file pone.0294572.s007.zip › S7_Appendix.pdf]

S7 Appendix Additional questions of Study 2

Support for different distribution principles *Note: Items were displayed in a randomized order. An additional option for "no answer/I don't know" was included.*

How important were the following considerations for your distributions? Please give your answers on a scale from 1 (not at all important) to 7 (very important).

- Each person should receive as much wood as they need.
- Each person should receive the wood they have chopped.
- Each person should receive the same amount of wood.

Political orientation *Note: An additional option for "no answer/I don't know" was included.*

In politics, one speaks of left-wing and right-wing. How would you describe your own political position in general? Where on a scale of 1 (left) to 7 (right) would you place yourself?

Sensitivity to cold *Note: An additional option for "no answer/I don't know" was included.*

On a scale from 1 (not at all sensitive to cold) to 7 (very sensitive to cold), how sensitive are you to cold?
