# Supplementary material for: Winter is coming: How laypeople think about different kinds of needs
Source: PLoS One. 2023 Nov 27;18(11):e0294572. doi: 10.1371/journal.pone.0294572 (PMC10681262; doi:10.1371/journal.pone.0294572)
Supplement: S1 Table — (ZIP) [file pone.0294572.s008.zip › S8_Table.pdf]

S8 Table Sample of Study 1 by gender, age, and income

Gender		Age		Income Interval[a]	
Group	Share	Group	Share	Group	Share
Female	50.0	18 – 29	21.0	[0, 1100)	16.0
Male	50.0	30 – 39	18.0	[1100, 1500)	23.0
		40 – 49	19.0	[1500, 2000)	23.0
		50 – 59	24.0	[2000, 2600)	19.0
		60 – 69	18.0	[2600, ∞)	19.0

Share in percent. $n = 100$. [a] Equivalent household net income.
