# Supplementary material for: Winter is coming: How laypeople think about different kinds of needs
Source: PLoS One. 2023 Nov 27;18(11):e0294572. doi: 10.1371/journal.pone.0294572 (PMC10681262; doi:10.1371/journal.pone.0294572)
Supplement: S3 Table — (ZIP) [file pone.0294572.s010.zip › S10_Table.pdf]

S10 Table Mean differences for Cases by Productivity Scenario

	Productivity Scenario					
	EPS		UPS			
Case (α, β)	$\overline{\Delta}_{\alpha,\beta}$	95% CI	$\overline{\Delta}_{\alpha,\beta}$	95% CI	Diff.	t
Sur – Sur	−14.8	$[-37.709, 8.080]$	−72.2	$[-132.921, -11.523]$	57.4	1.740*
Dec – Dec	0.0		−150.0	$[-213.765, -86.235]$	150.0	4.625***
Bel – Bel	9.3	$[-10.120, 28.725]$	−260.5	$[-339.553, -181.378]$	269.8	6.512***
Aut – Aut	−35.2	$[-84.039, 13.608]$	−225.5	$[-308.627, -142.432]$	190.3	3.882***
Sur – Aut	515.7	$[467.261, 564.119]$	388.5	$[331.488, 445.472]$	127.2	3.335***
Sur – Bel	425.4	$[380.155, 470.625]$	305.5	$[249.298, 361.682]$	119.9	3.259***
Dec – Aut	398.3	$[356.770, 439.790]$	251.0	$[196.734, 305.267]$	147.3	4.227***
Dec – Bel	295.3	$[255.957, 334.663]$	136.5	$[84.059, 188.941]$	158.8	4.750***
Sur – Dec	201.5	$[163.081, 239.939]$	129.0	$[82.679, 175.321]$	72.5	2.363***
Bel – Aut	122.6	$[85.082, 160.099]$	−27.5	$[-81.641, 26.641]$	150.1	4.469***

The table reports the mean differences ($\overline{\Delta}_{\alpha,\beta}$) of Paired Cases (upper part) and Mixed Cases (lower part) by Productivity Scenario as well as results of two-tailed Welch's t-tests. Significance levels: $^{*}\,p < 0.10$, $^{**}\,p < 0.05$, $^{***}\,p < 0.01$.
