# Supplementary material for: Winter is coming: How laypeople think about different kinds of needs
Source: PLoS One. 2023 Nov 27;18(11):e0294572. doi: 10.1371/journal.pone.0294572 (PMC10681262; doi:10.1371/journal.pone.0294572)
Supplement: S4 Table — (ZIP) [file pone.0294572.s011.zip › S11_Table.pdf]

S11 Table Percentage deviation of the share that Person A receives from the share that Person A contributed for Paired Cases by Productivity Scenario

	Productivity Scenario			
	EPS		UPS	
Case	Mean %	Std. Dev.	Mean %	Std. Dev.
Sur. – Sur.	0.741	4.279	10.556	4.538
Dec. – Dec.	0.000	0.000	9.000	4.678
Bel. – Bel.	0.698	3.196	6.791	5.276
Aut. – Aut.	1.765	8.867	7.489	6.037

The table reports means of absolute percentage deviations of the share that Person A receives from the share that Person A contributed for Paired Cases by Productivity Scenario.
