# Supplementary material for: Winter is coming: How laypeople think about different kinds of needs
Source: PLoS One. 2023 Nov 27;18(11):e0294572. doi: 10.1371/journal.pone.0294572 (PMC10681262; doi:10.1371/journal.pone.0294572)
Supplement: S5 Table — (ZIP) [file pone.0294572.s012.zip › S12_Table.pdf]

S12 Table Margins (Relative Need Evaluations) of Model (I)

Mixed Case α,β	Rel. Eval. $\overline{\Delta}_{\alpha,\beta}$	Wald Test χ^2
Sur – Aut	465.1***	$\overline{\Delta}_{Sur,Aut} = \overline{\Delta}_{Sur,Bel}$
	(21.9)	22.58***
Sur – Bel	372.1***	$\overline{\Delta}_{Sur,Aut} = \overline{\Delta}_{Sur,Dec}$
	(21.8)	232.89***
Sur – Dec	167.0***	$\overline{\Delta}_{Sur,Bel} = \overline{\Delta}_{Sur,Dec}$
	(21.8)	110.80***
Dec – Aut	330.8***	$\overline{\Delta}_{Dec,Aut} = \overline{\Delta}_{Dec,Bel}$
	(21.8)	33.09***
Dec – Bel	218.7***	
	(21.8)	
Bel – Aut	48.9**	
	(21.8)	
		joint
		277.45***

The table reports the margins (predicted means of relative need evaluations) $\overline{\Delta}_{\alpha,\beta}$ estimated by Tobit regression, see Table 5, Model (I). First row: mean; second row: standard error in parentheses. Margins significantly different from zero are marked with asterisks. χ^2 of a Wald test on the equality of two margins. Significance levels: $^{*}\,p < 0.10$, $^{**}\,p < 0.05$, $^{***}\,p < 0.01$. Wald tests with Bonferroni correction.
