# Supplementary material for: Winter is coming: How laypeople think about different kinds of needs
Source: PLoS One. 2023 Nov 27;18(11):e0294572. doi: 10.1371/journal.pone.0294572 (PMC10681262; doi:10.1371/journal.pone.0294572)
Supplement: S6 Table — (ZIP) [file pone.0294572.s013.zip › S13_Table.pdf]

S13 Table Margins (Relative Need Evaluations) by Productivity Scenario and Frame

Mixed Case α, β	Rel. Eval. $\overline{\Delta}_{\alpha,\beta}$	Wald Test χ^2	Rel. Eval. $\overline{\Delta}_{\alpha,\beta}$	Wald Test χ^2	Wald Test χ^2
Model (II)	Equal Productivity		Unequal Productivity		Eq. vs. Uneq.
Sur – Aut	532.3***	$\overline{\Delta}_{Sur,Aut} = \overline{\Delta}_{Sur,Bel}$	397.5***	$\overline{\Delta}_{Sur,Aut} = \overline{\Delta}_{Sur,Bel}$	$\overline{\Delta}^{Equal}_{Sur,Aut} = \overline{\Delta}^{Unequal}_{Sur,Aut}$
	(25.6)	13.75***	(25.4)	10.53***	25.31***
Sur – Bel	432.9***	$\overline{\Delta}_{Sur,Aut} = \overline{\Delta}_{Sur,Dec}$	310.9***	$\overline{\Delta}_{Sur,Aut} = \overline{\Delta}_{Sur,Dec}$	$\overline{\Delta}^{Equal}_{Sur,Bel} = \overline{\Delta}^{Unequal}_{Sur,Bel}$
	(25.4)	151.73***	(25.3)	100.26***	20.95***
Sur – Dec	203.1***	$\overline{\Delta}_{Sur,Bel} = \overline{\Delta}_{Sur,Dec}$	130.7***	$\overline{\Delta}_{Sur,Bel} = \overline{\Delta}_{Sur,Dec}$	$\overline{\Delta}^{Equal}_{Sur,Dec} = \overline{\Delta}^{Unequal}_{Sur,Dec}$
	(25.4)	74.51***	(25.3)	45.88***	7.41**
Dec – Aut	405.0***	$\overline{\Delta}_{Dec,Aut} = \overline{\Delta}_{Dec,Bel}$	256.2***	$\overline{\Delta}_{Dec,Aut} = \overline{\Delta}_{Dec,Bel}$	$\overline{\Delta}^{Equal}_{Dec,Aut} = \overline{\Delta}^{Unequal}_{Dec,Aut}$
	(25.3)	15.95***	(25.3)	19.53***	31.17***
Dec – Bel	298.6***		138.6***		$\overline{\Delta}^{Equal}_{Dec,Bel} = \overline{\Delta}^{Unequal}_{Dec,Bel}$
	(25.3)		(25.3)		36.23***
Bel – Aut	123.7***		−26.1		$\overline{\Delta}^{Equal}_{Bel,Aut} = \overline{\Delta}^{Unequal}_{Bel,Aut}$
	(25.3)		(25.3)		31.85***
		joint		joint	joint
		176.11***		124.04***	152.91***
Model (III)	Equal Productivity		Unequal Productivity		Eq. vs. Uneq.
Sur – Aut	546.2***	$\overline{\Delta}_{Sur,Aut} = \overline{\Delta}_{Sur,Bel}$	396.2***	$\overline{\Delta}_{Sur,Aut} = \overline{\Delta}_{Sur,Bel}$	$\overline{\Delta}^{Equal}_{Sur,Aut} = \overline{\Delta}^{Unequal}_{Sur,Aut}$
	(24.6)	13.80***	(25.0)	10.53***	27.51***
Sur – Bel	440.0***	$\overline{\Delta}_{Sur,Aut} = \overline{\Delta}_{Sur,Dec}$	305.3***	$\overline{\Delta}_{Sur,Aut} = \overline{\Delta}_{Sur,Dec}$	$\overline{\Delta}^{Equal}_{Sur,Bel} = \overline{\Delta}^{Unequal}_{Sur,Bel}$
	(24.5)	140.48***	(24.4)	86.92***	22.42***
Sur – Dec	208.3***	$\overline{\Delta}_{Sur,Bel} = \overline{\Delta}_{Sur,Dec}$	131.4***	$\overline{\Delta}_{Sur,Bel} = \overline{\Delta}_{Sur,Dec}$	$\overline{\Delta}^{Equal}_{Sur,Dec} = \overline{\Delta}^{Unequal}_{Sur,Dec}$
	(24.4)	66.59***	(24.4)	37.63***	7.37**
Dec – Aut	412.6***	$\overline{\Delta}_{Dec,Aut} = \overline{\Delta}_{Dec,Bel}$	252.1***	$\overline{\Delta}_{Dec,Aut} = \overline{\Delta}_{Dec,Bel}$	$\overline{\Delta}^{Equal}_{Dec,Aut} = \overline{\Delta}^{Unequal}_{Dec,Aut}$
	(24.5)	16.61***	(24.4)	20.72***	31.92***
Dec – Bel	296.9***		123.0***		$\overline{\Delta}^{Equal}_{Dec,Bel} = \overline{\Delta}^{Unequal}_{Dec,Bel}$
	(24.4)		(24.4)		37.65***
Bel – Aut	135.2***		−23.0		$\overline{\Delta}^{Equal}_{Bel,Aut} = \overline{\Delta}^{Unequal}_{Bel,Aut}$
	(24.4)		(24.4)		31.29***
		joint		joint	joint
		164.01***		110.61***	158.15***
Model (IV)	Avoidance		Enablement		Avoid. vs. Enable.
Sur – Aut	481.1***	$\overline{\Delta}_{Sur,Aut} = \overline{\Delta}_{Sur,Bel}$	449.3***	$\Delta_{Sur,Aut} = \Delta_{Sur,Bel}$	$\overline{\Delta}^{Avoid}_{Sur,Aut} = \overline{\Delta}^{Enable}_{Sur,Aut}$
	(30.9)	14.28***	(30.9)	8.68**	0.54
Sur – Bel	376.7***	$\overline{\Delta}_{Sur,Aut} = \overline{\Delta}_{Sur,Dec}$	367.5***	$\Delta_{Sur,Aut} = \Delta_{Sur,Dec}$	$\overline{\Delta}^{Avoid}_{Sur,Bel} = \overline{\Delta}^{Enable}_{Sur,Bel}$
	(30.8)	117.75***	(30.8)	115.46***	0.04
Sur – Dec	181.7***	$\overline{\Delta}_{Sur,Bel} = \overline{\Delta}_{Sur,Dec}$	152.2***	$\Delta_{Sur,Bel} = \Delta_{Sur,Dec}$	$\overline{\Delta}^{Avoid}_{Sur,Dec} = \overline{\Delta}^{Enable}_{Sur,Dec}$
	(30.8)	50.22***	(30.8)	61.00***	0.46
Dec – Aut	341.5***	$\overline{\Delta}_{Dec,Aut} = \overline{\Delta}_{Dec,Bel}$	320.0***	$\Delta_{Dec,Aut} = \Delta_{Dec,Bel}$	$\overline{\Delta}^{Avoid}_{Dec,Aut} = \overline{\Delta}^{Enable}_{Dec,Aut}$
	(30.8)	22.67***	(30.8)	11.43***	0.24
Dec – Bel	210.5***		226.9***		$\overline{\Delta}^{Avoid}_{Dec,Bel} = \overline{\Delta}^{Enable}_{Dec,Bel}$
	(30.8)		(30.8)		0.14
Bel – Aut	69.5**		28.32		$\overline{\Delta}^{Avoid}_{Bel,Aut} = \overline{\Delta}^{Enable}_{Bel,Aut}$
	(30.8)		(30.8)		0.89
		joint		joint	joint
		144.20***		134.99***	3.11

The table reports the margins (predicted means of relative need evaluations) $\overline{\Delta}_{\alpha,\beta}$ estimated by Tobit regression, see Table 5, Models (II)–(IV). First row: mean, second row: standard error in parentheses. Margins significantly different from zero are marked with asterisks. χ^2 of a Wald test on the equality of two margins. Significance levels: $^{*}\,p < 0.10$, $^{**}\,p < 0.05$, $^{***}\,p < 0.01$. Wald tests with Bonferroni correction.
