# Supplementary material for: Winter is coming: How laypeople think about different kinds of needs
Source: PLoS One. 2023 Nov 27;18(11):e0294572. doi: 10.1371/journal.pone.0294572 (PMC10681262; doi:10.1371/journal.pone.0294572)
Supplement: S7 Table — (ZIP) [file pone.0294572.s014.zip › S14_Table.pdf]

S14 Table Control Variables for Study 2

	(4)
Age	-1.207
$\{\sharp years\}$	(1.082)
Gender	10.378
$\{0 = female, 1 = male\}$	(34.03)
Household Net Income	-0.00124
$\{euros\}$	(0.000814)
Importance Need	23.07^{**}
$\{1, \ldots, 7\}$	(11.169)
Importance Productivity	-78.36^{***}
$\{1, \ldots, 7\}$	(9.372)
Importance Equality	-17.282^{*}
$\{1, \ldots, 7\}$	(9.075)
Political Attitude	11.330
$\{1, \ldots, 7\}$	(14.952)
Sensitivity to Cold	-2.860
$\{1, \ldots, 7\}$	(10.485)

The table reports the covariates of a Tobit random-effects panel regressions with robust standard errors (see Model (III) in Table 5). First row: coefficients, second row: standard errors in parentheses. Significance levels: $^{*}\,p < 0.10$, $^{**}\,p < 0.05$, $^{***}\,p < 0.01$.
